# Noise-Aware Machine Learning Accelerates Development of High-Latent-Heat Cu-Al-Ni Shape Memory Alloys for Thermal Management

**DOI:** 10.3390/ma19132802

**Published:** 2026-07-01

**Authors:** Donghua Zhou, Xiaohua Tian, Hongxing Li, Xiangyu Tong, Mingchao Zhang, Jieyu Meng, Yefei Wang, Wenbin Zhao, Jian Li, Changlong Tan

**Affiliations:** 1 School of Electrical and Electronic Engineering, Harbin University of Science and Technology, Harbin 150080, China; 2 School of Materials Science and Chemical Engineering, Harbin University of Science and Technology, Harbin 150080, China

**Keywords:** noise-aware machine learning, Cu-Al-Ni shape memory alloys, latent heat, martensite transformation temperature

## Abstract

Cu-Al-Ni shape memory alloys (SMAs) are promising solid–solid phase-change materials (PCMs) for transient thermal management. Data-driven screening for high-latent-heat (Δ*H*) Cu-Al-Ni PCMs across the vast compositional space is efficient, but predictive accuracy and screening reliability degrade when noisy experimental data are used. A noise-aware machine learning strategy was applied to accelerate the discovery of high-Δ*H* Cu-Al-Ni alloys with martensite start temperature (*M_s_*) within the 100–200 °C range from noisy experimental datasets. The optimal noise level was estimated by minimizing the prediction error of the noise-aware Kriging model. The application of this strategy led to the discovery of four Cu-Al-Ni alloys with *M_s_* ranging from 125 to 163 °C and Δ*H* ranging from 9.27 to 9.86 J/g. The best-performing Cu_84_Al_13_Ni_3_ (wt.%) alloy achieved *M_s_* = 163 °C, Δ*H* = 9.86 J/g, thermal conductivity of 102 W·m^−1^·K^−1^ and figure of merit of 7272 × 10^6^ J^2^ K^−1^ s^−1^ m^−4^. Its Δ*H* exceeds the previous highest Cu-Al-Ni Δ*H* in the 100–200 °C window by 11.8%, while its *FOM* exceeds the previous highest Cu-Al-Ni *FOM* by 33.75% and represents the highest value among the surveyed PCMs within the 100–200 °C range. After 100 thermal cycles, Δ*H* decreased by 0.158 J/g and *M_s_* shifted by 0.9 °C, demonstrating good thermal cycling stability.

## 1. Introduction

As the power densities of high-power electronic devices continue to increase, rapid temperature spikes caused by transient high heat flux have become a critical bottleneck limiting device performance and reliability [1]. Solid–solid phase-change materials (PCMs), which avoid liquid leakage, exhibit negligible volumetric change, and are non-toxic and non-corrosive, have emerged as important candidate materials for electronics thermal management [2,3,4,5]. However, traditional solid–solid PCMs inherently suffer from low thermal conductivity, severely limiting their heat transfer capacity and response speed, and making it difficult to meet the transient cooling demands of high-power devices. By contrast, shape memory alloys (SMAs) can efficiently absorb and release large amounts of latent heat (Δ*H*) through reversible martensite phase transformations and thus offer a new approach to transient thermal management in high-power electronics [2,4]. Current research on SMAs for thermal management has focused primarily on Ni-Ti-based alloys [2,3,4,6,7,8], but these alloys have thermal conductivities typically below 23 W·m^−1^·K^−1^ (see Table S8), which is insufficient for rapid heat dissipation under high-heat-flux conditions. In contrast, Cu-Al-based SMAs, which are composed of highly thermally conductive elements such as Cu (385 W·m^−1^·K^−1^) and Al (218 W·m^−1^·K^−1^), stand out by virtue of their superior thermal conductivity [9]. Among these, Cu-Al-Ni SMAs combine a suitable transformation temperature, good thermal cycling stability, and low thermal hysteresis, while possessing significantly higher thermal conductivity than conventional Ni-Ti SMAs. They have demonstrated unique advantages in applications requiring fast heat transfer, such as power modules, RF devices, and automotive power electronics [9,10,11]. Therefore, developing a Cu-Al-Ni SMA with high Δ*H* in the 100–200 °C temperature range is of great significance for overcoming the thermal management bottleneck in high-power electronic devices.

However, conventional Cu-Al-Ni shape memory alloys exhibit relatively low Δ*H*, which makes them unable to meet the thermal management requirements of high-power electronic devices. Therefore, the development of high-latent-heat compositions faces two core challenges, as detailed below. First, the martensitic transformation behavior of Cu-Al-Ni SMAs is extremely sensitive to composition: minute compositional variations, elemental segregation, or precipitate formation can markedly alter the martensite start temperature (*M_s_*), latent heat, and the width of the thermal hysteresis [12]. Second, the compositional space of the ternary Cu-Al-Ni system is enormous (on the order of 10^6^ possible combinations), and traditional trial-and-error experimentation is prohibitively costly and time-consuming. It is difficult to precisely pinpoint the optimal composition that satisfies the target transformation temperature window and high-Δ*H* requirements within such a vast space.

In recent years, data-driven machine learning methods combined with Bayesian optimization have provided an effective avenue to address the above challenges. These approaches can capture complex non-linear relationships between composition, structure, and properties even with limited samples, and accelerate materials discovery through iterative active sampling [13,14,15,16,17,18]. Their key advantage is that a trained model can rapidly predict material performance without exhaustive experimentation, providing crucial guidance for efficiently screening high-performance materials in extremely large composition spaces [19,20].

However, the predictive accuracy of machine learning models depends strongly on the quality and consistency of the training data, while measurements of Δ*H* and *M_s_* are subject to uncertainties arising from variations in sample preparation, baseline correction, instrument drift, and property changes during repeated thermal cycling. These factors introduce significant inherent noise into the data [2,4,5,17]. Moreover, the literature data often report only representative measurement values without systematic repeat statistics, making it difficult to accurately quantify the noise level. Training a model directly on high-noise, sparse data can cause the model to mistake noise for real patterns, leading to large prediction errors. Furthermore, when extrapolating beyond well-sampled compositional regions, the model may produce unreliable predictions and thereby mislead active-learning decisions during the identification of optimal compositions [21,22,23]. Currently, small and noisy datasets have become a key bottleneck limiting data-driven material development.

To tackle the uncertainty arising from data noise and sparse sampling, the current solutions generally fall into two categories. The first category focuses on enhancing model quality and robustness to mitigate the negative impact of uncertainty. For example, Zhang et al. improved noise and bias tolerance by employing ensemble learning to aggregate outputs from multiple models, achieving more stable predictive performance [21]. Ojih et al. used Lasso/Ridge regularization to curb overfitting and improve generalization in data-limited screening [24]. Although these strategies can significantly improve prediction accuracy and stability, observational noise and epistemic uncertainty remain unavoidable. Uncertainty cannot be completely eliminated, and thus predictive reliability and iterative efficiency remain limited. The second category explicitly quantifies and leverages uncertainty in experimental design to optimize sampling decisions. By using a utility function to evaluate the information value of candidate samples, the most informative samples are prioritized for experimentation, strategically augmenting the dataset to reduce uncertainty due to data scarcity [15,18]. For example, Jo et al. quantified model uncertainty via the standard deviation and preferentially sampled high-uncertainty candidates in each iteration, thereby improving predictions and reducing overall uncertainty [25]. Xue et al. combined mathematical inference with global optimization to balance exploitation and exploration. Starting from an initial training set of 22 samples, they developed an SMA with only 1.84 K thermal hysteresis after six rounds of experiments [17]. However, the above methods mostly address the epistemic uncertainty arising from sparse sampling. If observational noise is non-negligible and not explicitly modeled, sampling decisions may be perturbed by noise and deviate from the optimum. For Cu-Al-Ni shape memory alloys, this issue is particularly critical because publicly available data remain limited, and observational noise can arise from cross-study differences in alloy preparation, heat-treatment procedures, and testing protocols. We hypothesize that a noise-aware machine learning strategy can improve model prediction accuracy and enhance candidate-screening effectiveness by explicitly accounting for observational noise under small-sample and noisy Cu-Al-Ni experimental data conditions.

The contribution of this work lies in applying a noise-aware machine learning strategy to the reliable design of high-Δ*H* Cu-Al-Ni shape memory alloys with *M_s_* values within the 100–200 °C temperature window under small-sample and noisy experimental data conditions. This contribution is further demonstrated through experimental validation of the effectiveness of the strategy and the thermal-management performance of the designed alloys, ultimately leading to the identification of a newly designed Cu-Al-Ni composition that exhibited the highest figure of merit (*FOM*) among the surveyed PCMs within the 100–200 °C range. The effective observational noise level was estimated by minimizing the error of the noise-aware Kriging model, after which an optimized noise-aware Kriging model was constructed to establish quantitative relationships between alloy descriptors and target properties. The effect of explicitly incorporating observational noise on prediction performance was then evaluated through comparison with random forest regression (RFR), extreme gradient boosting regression (XGBR), light gradient boosting regression (LGBR) and the standard Kriging model. To further assess the generalization ability of the optimized Δ*H* model for unseen Cu-Al-Ni compositions, predicted Δ*H* values were compared with the corresponding reported experimental values using an independent external validation set excluded from the original training dataset. Using the optimized models, *M_s_* and Δ*H* were predicted across the virtual Cu-Al-Ni compositional space. Then, candidate alloys were retained if their predicted *M_s_* values fell within 100–200 °C. These candidates were further ranked according to their predicted Δ*H* values and the sequential Kriging optimization (*SKO*) acquisition scores calculated from the noise-aware Δ*H* model. Finally, selected alloys were fabricated and characterized to validate the screening results and evaluate their thermal management performance. Overall, this study applies and validates a noise-aware machine learning strategy suitable for small-sample and high-noise material data conditions and provides a data-driven route toward the development of Cu-Al-Ni shape memory alloys for transient thermal management applications.

## 2. Research Methods

**Figure 1:**
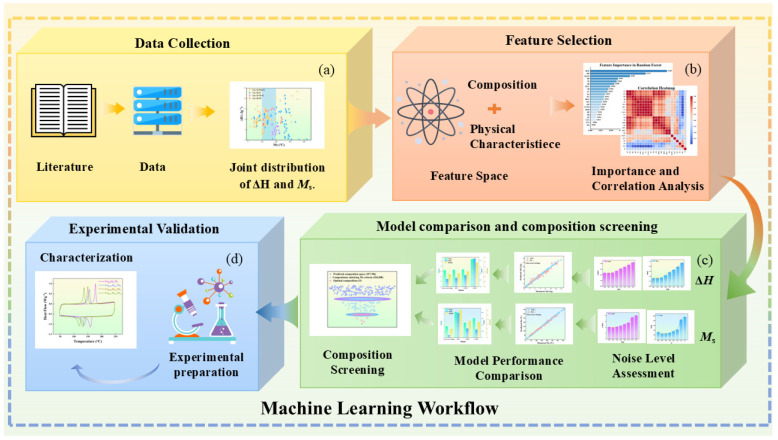
Schematic illustration of the noise-aware machine learning strategy for developing high-latent-heat (∆*H*) Cu-Al-Ni phase-change materials (PCMs) with martensite start temperature (*M_s_*) in the 100–200 °C range. (**a**) Data collection. (**b**) Feature selection. (**c**) Model comparison and composition screening. (**d**) Experimental validation.

As shown in Figure 1, a workflow comprising data construction, feature engineering, model optimization and prediction, and experimental validation was applied to predict the Δ*H* and *M_s_* of Cu-Al-Ni PCMs. Within this workflow, noise-aware and noise-ignoring strategies were systematically compared, and the optimal model was selected for performance prediction across the Cu-Al-Ni compositional space.

### 2.1. Dataset Construction

To establish predictive models for Cu-Al-Ni SMAs, the literature data on *M_s_* and Δ*H* were collected from the Cu-Al-Ni subsystem, as well as related Cu-Al-based alloy systems. The reliability of machine learning modeling depends strongly on data quality and sample consistency. For Cu-Al-based SMAs reported in the literature, substantial variations commonly exist among different studies in fabrication routes, heat-treatment conditions, and characterization protocols. Such non-compositional factors can markedly affect transformation behavior and thermodynamic response [12]. If heterogeneous data are used directly for model training without appropriate control, the model may misinterpret fluctuations arising from experimental inconsistency as intrinsic composition–property relationships, thereby compromising predictive accuracy and generalization capability. Because Cu-Al-Ni data remain limited for both *M_s_* and Δ*H* modeling, using this subsystem alone would produce a small and sparsely distributed training set, thereby limiting model stability and generalization capability. Considering that Cu-Al-Ni alloys belong to the broader family of Cu-Al-based SMAs and share certain composition–property similarities with related Cu-Al-based alloy systems, the *M_s_* and Δ*H* datasets were expanded to include these related systems, with the aim of increasing the sample size and improving model robustness. This data expansion does not assume identical transformation behavior or latent-heat characteristics across all Cu-Al-based subsystems. Rather, the inclusion of related alloy systems was intended to help the models learn more general composition–property trends while retaining the final prediction and screening within the target Cu-Al-Ni design space. Finally, targeted experimental validation was performed to assess the applicability of this data-expansion strategy to Cu-Al-Ni alloys.

To reduce the confounding effects introduced by non-compositional factors and improve the reliability of capturing composition–property relationships, the collected literature data were systematically screened according to the following criteria:

(i) Only data from samples prepared by vacuum arc melting were retained.
(ii) Only data from samples subjected to homogenization or solution treatment were included.
(iii) Only phase transformation data measured by differential scanning calorimetry (DSC) were accepted.
(iv) For compositions measured by transmission electron microscopy (TEM) or scanning electron microscopy with energy-dispersive X-ray spectroscopy (SEM-EDS), any dataset in which the elemental atomic fractions did not sum to (100 ± 0.1) at. % was discarded.

### 2.2. Feature Engineering

To improve model generalization capability and interpretability, multi-dimensional materials descriptors were introduced to represent alloy compositions. In total, 22 candidate features were constructed, covering three categories of information: (i) intrinsic elemental physical properties (e.g., atomic radius, valence electron concentration); (ii) thermodynamic/thermophysical parameters related to the martensitic transformation (e.g., boiling point, thermal expansion coefficient); and (iii) key processing parameters (e.g., solution treatment temperature). Definitions of all descriptors are summarized in Supplementary Table S3. For alloy-level features, a weighted average of the elemental properties (weighted by atomic percentage) was used: for a given feature, $X=\sum_{i}f_{i}X^{\left(i\right)}$, where $X^{\left(i\right)}$ is the property value for element *i*, $f_{i}$ is its atomic fraction in the alloy, and X is the resulting weighted feature. Using all 22 features directly for model training would increase the computational cost and the risk of overfitting. Therefore, a hybrid feature selection strategy combining random forest (RF) importance ranking and a Pearson correlation threshold was employed to reduce redundancy while preserving critical information. The specific procedure is as follows:

(i) Importance ranking: Separate RFR models were trained for Δ*H* and *M_s_* prediction, and the 22 features were ranked according to their relative importance.
(ii) Correlation analysis: Pearson correlation coefficients were calculated between all possible feature pairs. The resulting correlation matrix was then visualized as a heatmap to identify highly correlated descriptors and evaluate potential redundancy among the features.
(iii) Redundancy elimination: For each highly correlated feature pair (|r| > 0.9), the feature with the higher RFR-derived importance was retained, while the less important feature was removed to reduce redundancy.

Here, the Pearson correlation coefficient between two variables X and Y is defined as $\rho_{XY}=\;\frac{Cov(X,Y)}{\sqrt{D(X)}\sqrt{D(Y)}}=\frac{E[(X-EX)(Y-EY)]}{\sqrt{D(X)}\sqrt{D(Y)}}$, where *E* denotes expectation and D denotes variance.

### 2.3. Model Optimization and Prediction

For the continuous target variables Δ*H* and *M_s_*, five models were constructed and compared: the noise-aware Kriging model; the standard Kriging model without an observation-noise term; and three deterministic regression models, including RFR, XGBR, and LGBR. Their performance was evaluated using multiple metrics, and the best-performing model was selected for compositional-space prediction. The models are detailed below:

**Noise-aware Kriging model:** Noise-aware Kriging (also known as Gaussian process regression with noise) has been proven to be effective for improving material property prediction and optimization efficiency under small-sample, high-noise conditions [26]. In the noise-aware Kriging formulation, each experimentally measured or literature-reported *M_s_* or ∆*H* value is treated as a noisy observation. The corresponding underlying true value refers to the material property response under a specified material state after excluding observational errors. This relationship is expressed as $\tilde{y_{i}}\;={\;y}_{i}\;+\;\varepsilon_{i}$, where $\tilde{y_{i}}$ is the observed value; $y_{i}$ is the underlying true value; and $\varepsilon_{i}$ represents a single realization of an observational-noise random variable assumed to follow a Gaussian distribution $N\;(0,\;\tau^{2})$, with *τ*^2^ denoting the noise variance. Here, observational noise represents the deviation between the reported value and the underlying response, mainly reflecting effective observational uncertainty associated with measurement errors; differences in testing or data-processing methods; repeated-measurement scatter; and variations among different data sources, including differences in instrument conditions, testing protocols, and sample states. To account for this noise, a diagonal noise matrix Δ is introduced into the Gaussian-process covariance structure. The diagonal terms of Δ represent the noise variance *τ*^2^, and the covariance matrix is therefore modified from *K* to *K +* Δ. In this way, observational noise is explicitly incorporated into the model-output uncertainty estimation. The predicted mean *u*(*x*) and predictive variance *s*^2^(*x*) for a candidate point *x* are given by analytical expressions, as shown in Equations (S1) and (S2).

Since the true noise level of the Cu-Al-Ni dataset is unknown, candidate noise levels were defined as scaled fractions of the output-value range, as described in Equation (S3). The noise-level parameter prefactor *Φ* was treated as a hyperparameter and searched over a broad range. For each candidate *Φ*, leave-one-out cross-validation (*LOO-CV*) was performed to calculate the mean squared error (*MSE*) and continuous ranked probability score (*CRPS*). The *Φ* value that minimized both metrics was selected as the optimal noise-level prefactor, and the corresponding effective observation-noise-level *τ* was calculated according to Equation (S3) for training the final model (see Equations (S4) and (S5)). This procedure enabled the effective noise level of the existing literature-derived experimental dataset to be quantitatively estimated without additional repeated experiments. After this optimization, the final noise-aware Kriging model was established using this calibrated noise level. A noise-enhanced *SKO* acquisition function was then applied during virtual compositional screening to rank candidate alloys [27], with its explicit form provided in Equation (S6). By incorporating the predicted mean *u*(*x*), predictive variance *s*^2^(*x*), and calibrated observation-noise variance *τ*^2^ into the *SKO* acquisition function, the screening process considered not only the predicted property values, but also the model uncertainty, represented by *s*^2^(*x*), and the influence of experimental observation noise, represented by *τ*^2^, on decision reliability. The Kriging models were implemented in the R statistical computing environment using the DiceKriging package developed by Roustant et al. [28].

**Noise-ignoring baseline models:** For comparison, four noise-ignoring machine learning baseline models were constructed, including a standard Kriging model and three deterministic regression models. The standard Kriging model was obtained by setting the noise-level prefactor *Φ* = 0, corresponding to a noise-free Gaussian process regression baseline. RFR, XGBR, and LGBR were further constructed as deterministic machine learning baselines. During training, the literature data were treated as deterministic observations. The deterministic regression models were implemented in Python 3.9.18 with standard libraries.

(i) RFR: An ensemble bagging algorithm that aggregates many decision trees via voting to reduce overfitting, offering strong noise robustness on small-sample datasets [29].
(ii) XGBR: An improved gradient boosting method that introduces regularization to prevent overfitting, and has demonstrated excellent accuracy in material property regression tasks [30].
(iii) LGBR: A gradient boosting method using histogram-based optimization and gradient-based one-side sampling, which trains quickly with low memory usage, making it suitable for small-sample material datasets [31].

All models used an 80:20 random split for the training and test sets. For both Δ*H* and *M_s_* prediction tasks, model performance on the test set was evaluated using the test-set coefficient of determination (*Test_R*^2^), mean absolute error (*MAE*), *MSE*, and root mean squared error (*RMSE*). In addition, the five-fold cross-validated coefficient of determination (*CV_R*^2^) was calculated to assess generalization stability under different data splits. For the Kriging model, the predicted mean was taken as the point prediction, and *CRPS* was used to evaluate the quality of its probabilistic predictions (i.e., uncertainty quantification). The coefficient of determination *R*^2^ (range: 0–1) represents the fraction of variance in the target explained by the model, with values closer to 1 indicating better agreement between predictions and measurements. The mean absolute error (*MAE*), *MSE*, and *RMSE* quantify predictive deviation, with lower values indicating higher accuracy. The formulas and definitions of these metrics are provided in Equations (S7)–(S10).

After the optimal model had been identified, the model was applied to a virtual Cu-Al-Ni compositional space for candidate composition screening. To narrow the design space and ensure that the candidates fell within the composition range capable of undergoing martensitic transformation, the following constraints were imposed based on statistical analysis of the literature data and experimental experience. The virtual alloy composition was expressed as Cu*_x_*Al*_y_*Ni*_z_*, where *x*, *y*, and *z* denote the atomic percentages of Cu, Al, and Ni, respectively. The compositional limits were set as 67.22% ≤ *x* ≤ 75.52%, 20.76% ≤ *y* ≤ 28.46%, 2.72% ≤ *z* ≤ 5.42%, and *x* + *y* + *z* = 100%. A compositional step size of 0.01 at.% was adopted to construct a dense virtual sampling grid for response-surface evaluation, enabling high-resolution exploration of the composition–property landscape. This step size was used only for virtual compositional screening and does not represent the experimental compositional precision. All valid virtual compositions satisfying these constraints were generated in Python, resulting in a total of 197,786 possible compositions. The trained model was then employed to predict Δ*H* and *M_s_* for each composition, and an acquisition function was used to rank and select candidate compositions for experimental validation.

### 2.4. Experimental Validation

High-purity elemental Cu, Al, and Ni (≥99.99%) were used as raw materials to prepare alloy ingots in a non-consumable vacuum arc melting furnace, with electromagnetic stirring applied during melting. To improve compositional homogeneity, each ingot was inverted and remelted five times. After cooling, the ingots were sealed in evacuated quartz tubes under argon, solution-treated at 900 °C for 30 min, then immediately water-quenched to room temperature. The heat-treated ingots were cut into two sample geometries: Φ3 × 1 mm disks were used for phase transformation characterization, thermal cycling tests, specific heat capacity measurements, and microstructural observation, whereas larger Φ12.63 × 2 mm disks were used for density and thermal diffusivity measurements.

DSC was used to measure the phase transformation temperatures, Δ*H*, and thermal cycling stability of the alloys. DSC tests were performed over the temperature range of 100–220 °C at a heating/cooling rate of 40 °C/min, using cyclic thermal scans. The martensite start temperature (*M_s_*), martensite finish temperature (*M_f_*), austenite start temperature (*A_s_*), and austenite finish temperature (*A_f_*) were determined from the DSC curves using the tangent-intercept method. Δ*H* was obtained by integrating the area under the endothermic/exothermic peaks of the DSC curves. To evaluate thermal cycling stability, each sample was subjected to 10 consecutive thermal cycles at 20 °C/min and 100 consecutive cycles at 40 °C/min. The DSC data were analyzed with Origin software to track changes in Δ*H* and *M_s_* as a function of cycle number.

The specific heat capacity (*C_p_*) was measured by the sapphire method using a TA Instruments DSC 2500, with a heating rate of 10 °C/min over a temperature range of 100–220 °C. The sample density (*ρ*) was measured by the Archimedes drainage method. The thermal diffusivity (*α*) was measured via the laser flash method (Netzsch LFA 427). The thermal conductivity *k* was then calculated from the relation *k* = *C_p_*·*α*·*ρ*.

X-ray diffraction (XRD) and scanning electron microscopy (SEM) were used to analyze the phase constitution and microstructure of the samples. The sample surfaces were ground with SiC papers of progressively finer grit, then polished with finer media, and finally polished with a diamond paste on a cloth to obtain a scratch-free surface. A chemical etchant (FeCl_3_ 1 g + C_2_H_6_O 1 mL + H_2_O 5 mL) was applied for 5–10 s to reveal the microstructure. Variable-temperature XRD patterns were collected using a Bruker D8 Advance diffractometer (Cu *Kα* radiation) while heating the sample from 25 °C to 250 °C. An FEI TESCAN MIRA LMS SEM was used to observe the etched microstructure of the alloy.

## 3. Results and Discussion

### 3.1. Data Distribution

After screening, the final dataset comprised 164 data records for ∆*H* and 198 data records for *M_s_* (Tables S1 and S2). The dataset included Cu-Al-based alloy subsystems containing Mn, Ni, Ti, Mg, Fe, Cr, Zr, Hf, V, Ga, Nb, Sn, Te, and Co. Figure S1a shows that the Δ*H* dataset is unevenly distributed in the target-property space. Among the 164 Δ*H* data points, 86.0% are distributed within the range of 0–9 J/g, whereas samples with Δ*H* higher than 9 J/g account for only 14.0%. This indicates that the high-latent-heat region is underrepresented in the dataset, increasing the difficulty of screening high-performance candidate alloys. As shown in Figure S1b, the *M_s_* data are unevenly distributed across the transformation temperature range. The target operating window of 100–200 °C accounts for 44.4% of the *M_s_* dataset, higher than the fractions below 100 °C (26.3%) and above 200 °C (29.3%). This indicates relatively better data coverage in the target window, providing stronger support for model training and prediction within the temperature range of practical relevance. Figure S1c further shows that compositions simultaneously exhibiting high Δ*H* and *M_s_* values within the target range are limited. In addition, candidate selection for transient thermal management is constrained not only by Δ*H* and *M_s_*, but also by thermal conductivity. Although Cu-Al-Mn-X alloys can exhibit relatively high Δ*H* at lower transformation temperatures, their lower thermal conductivity limits their suitability for rapid heat-transport applications. In contrast, Cu-Al-Ni alloys exhibit substantially higher thermal conductivity, with reported values up to 101 W·m^−1^·K^−1^ [9], making them more suitable for transient thermal management applications. Therefore, although the model was trained on an expanded Cu-Al-based dataset to capture broader composition–property relationships, final screening was restricted to the Cu-Al-Ni subsystem within the 100–200 °C window, and this strategy was subsequently validated by targeted experiments on Cu-Al-Ni alloys.

### 3.2. Feature Selection

The feature selection results are shown in Figure S2. For Δ*H* prediction, the feature importance ranking indicates that heat of vaporization (*HOV*), melting point (*MP*), and heating/cooling rate (*RATE*) are the top contributors, followed by boiling point (*BP*), thermal expansion (*TE*), and specific heat (*SH*). This suggests that thermodynamic/thermophysical descriptors have a dominant influence on ∆*H*. Further correlation analysis was used to identify highly redundant features. For example, the covalent radius (*CR*) had Pearson correlation coefficients exceeding 0.93 with both the atomic Waber–Cromer pseudopotential radius (*DOR*) and the atomic radius (*AR*). Because the importance of *CR* (0.044) was higher than those of *DOR* (0.022) and *AR* (0.015), *DOR* and *AR* were removed, whereas *CR* was retained. Combined importance ranking and Pearson-correlation filtering yielded seven retained features for the Δ*H* model: *SH*, *TE*, *CR*, *HOF*, *BP*, thermal conductivity (*TC*), and *RATE*. Specifically, *SH* reflects heat capacity and vibrational entropy contributions. Since the latent heat of martensitic transformation essentially originates from the enthalpy difference between austenite and martensite and is closely associated with transformation entropy, *SH* provides thermodynamically relevant information for Δ*H* prediction. *TE* describes lattice anharmonicity and volume–strain coupling, which may affect lattice energy, elastic energy, and transformation barriers. *CR* reflects atomic-size mismatch and local lattice distortion; larger size mismatch may increase local strain and interfacial migration resistance, thereby influencing the transformation enthalpy during martensitic transformation. *HOF* and *BP* are associated with cohesive-energy and bond-strength scales. Stronger metallic bonding generally corresponds to a higher cohesive-energy scale and may indirectly affect Δ*H* by modifying the relative phase stability and free-energy difference between austenite and martensite. *TC* mainly represents electron/phonon thermal-transport behavior, whereas *RATE* characterizes the influence of DSC heating/cooling rate on the measured transformation enthalpy.

As shown in Figure S3, the feature importance ranking for *M_s_* prediction shows that *BP*, *TE*, and *SH* have the greatest influence on *M_s_*, followed by electron affinity (*EA*), molar volume (*MV*), and heat of fusion (*HOF*). This again highlights the significant role of thermodynamic/thermophysical features in phase transformation behavior. Redundancy among descriptors was further reduced using Pearson correlation analysis: for instance, *BP* and *HOV* had a correlation coefficient of 0.99534, and *BP*’s importance (0.25825) is much higher than *HOV*’s (0.06266), so *BP* was retained over *HOV* in this highly correlated pair. Similarly, *SH* showed correlation coefficients above 0.9 with density (*DE*), molar volume (*MV*), valence electron number (*VEN*), and average valence electron number (*CVEN*) (other features related to electron density and valence electron number). After considering both importance and correlation, *SH* was retained. The final feature set for *M_s_* prediction was simplified to *BP*, *TE*, *SH*, *CR*, *EA*, *HOF*, atomic number (*AN*), and *RATE*. Detailed feature information is provided in Table S4. Among these descriptors, *BP* is associated with elemental cohesive-energy and bond-strength scales, which may influence *M_s_* by modifying the relative stability and free-energy difference between austenite and martensite. *TE* reflects lattice anharmonicity, phonon softening, and volume–strain coupling. These lattice-related effects can change the elastic-energy contribution and the critical driving force required for martensitic transformation, thereby affecting the transformation temperature. *SH* is related to heat-capacity differences and entropy contributions, which determine the temperature dependence of the free-energy difference between the parent and martensitic phases and thus influence the temperature at which martensitic transformation starts. *CR* represents atomic-size mismatch and local lattice distortion; these effects may alter lattice compatibility, local strain energy, and interfacial resistance, thereby changing the undercooling required for transformation and affecting *M_s_*. *EA* and *AN* may affect *M_s_* through their influence on phase stability and electronic contributions to the free-energy balance. *HOF* is associated with phase-stability-related thermodynamic energy scales. *RATE* mainly reflects the influence of DSC heating/cooling rate on the measured *M_s_*.

### 3.3. Noise-Level Assessment

**Figure 2:**
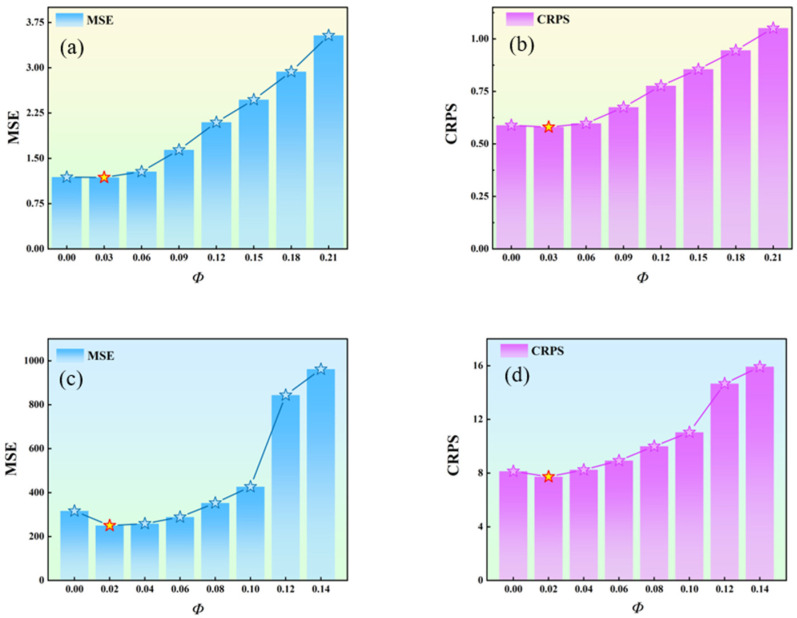
Effect of the input noise-level parameter prefactor *Φ* on predictive accuracy and uncertainty. (**a**) Mean squared error (*MSE*) on the ∆*H* dataset. (**b**) Continuous ranked probability score (*CRPS*) on the ∆*H* dataset. (**c**) *MSE* on the *M_s_* dataset. (**d**) *CRPS* on the *M_s_* dataset.

The effect of the preset noise level on model accuracy and uncertainty was evaluated to determine the optimal noise parameter (Figure 2). The noise standard deviation was parameterized as *τ* = [*Φ*(*y^max^* − *y^min^*)], where *Φ* is the input noise-level parameter prefactor.

For the Δ*H* dataset, the input noise-level prefactor *Φ* was varied over the range [0, 0.21]. As shown in Figure 2a,b, both *MSE* and *CRPS* first decrease and then increase with increasing *Φ*, reaching their minimum values simultaneously at *Φ* = 0.03. This indicates that *Φ* = 0.03 is the optimal noise-level parameter prefactor for the Δ*H* dataset. With *y^max^* = 13.99 J/g and *y^min^* = 0.11 J/g, the corresponding optimal noise standard deviation for Δ*H is τ* = [*Φ*(*y^max^* − *y^min^*)] = 0.417 J/g. For the *M_s_* dataset, Figure 2c,d shows that *MSE* and *CRPS* reach their minima at *Φ* = 0.02, identifying *Φ* = 0.02 as the optimal noise-level parameter prefactor. With *y^max^* = 360.54 °C, and *y^min^* = 1.3 °C, the corresponding optimal noise standard deviation was *τ* = [*Φ*(*y^max^* − *y^min^*)] = 7.18 °C. At this setting, the noise-aware Kriging model’s *LOO-CV MSE* for *M_s_* is 245, whereas that of the standard Kriging model is 316. In other words, the noise-aware Kriging achieves an *MSE* about 22.5% lower than the standard Kriging model for *M*_s_ prediction.

This non-monotonic dependence of *MSE* and *CRPS* on *Φ* reflects a trade-off between suppressing overfitting to noise-induced local variations and preserving intrinsic composition–property relationships. When *Φ* = 0, all observations are effectively treated as noise-free, so the model tends to overfit variations arising from experimental uncertainty. As *Φ* increases, observational noise is incorporated into the covariance structure, reducing sensitivity to such fluctuations and enabling the model to capture more robust composition–property trends. However, when *Φ* becomes excessively large, meaningful structural information is also suppressed, resulting in an oversmoothing surrogate response, underfitting, and increased prediction errors. Therefore, the optimal noise level is achieved when these two effects are best balanced.

Compared with Δ*H*, the performance gain achieved by noise-aware modeling was more pronounced for *M_s_*, indicating that explicit noise modeling tends to be more effective when the dataset itself exhibits greater variability.

### 3.4. Model Performance Comparison

**Figure 3:**
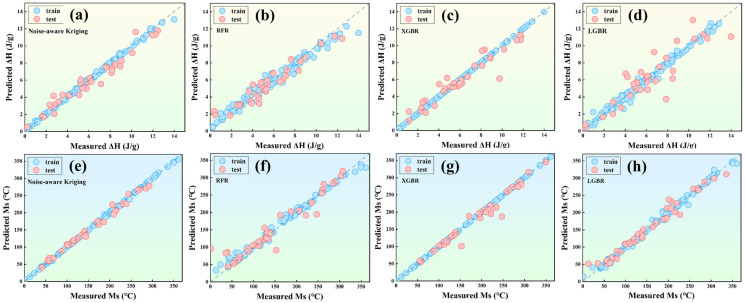
Predictive performance of different machine learning models on the training and test datasets. Blue and red symbols represent the training and test sets, respectively. The dashed 45° diagonal line denotes perfect agreement between predicted and measured values. Panels (**a**–**d**) compare predicted and measured Δ*H* values, while panels (**e**–**h**) compare predicted and measured *M_s_* values. (**a**) Noise-aware Kriging; (**b**) random forest regression (RFR); (**c**) extreme gradient boosting regression (XGBR); (**d**) light gradient boosting regression (LGBR); (**e**) noise-aware Kriging; (**f**) RFR; (**g**) XGBR; (**h**) LGBR.

**Figure 4:**
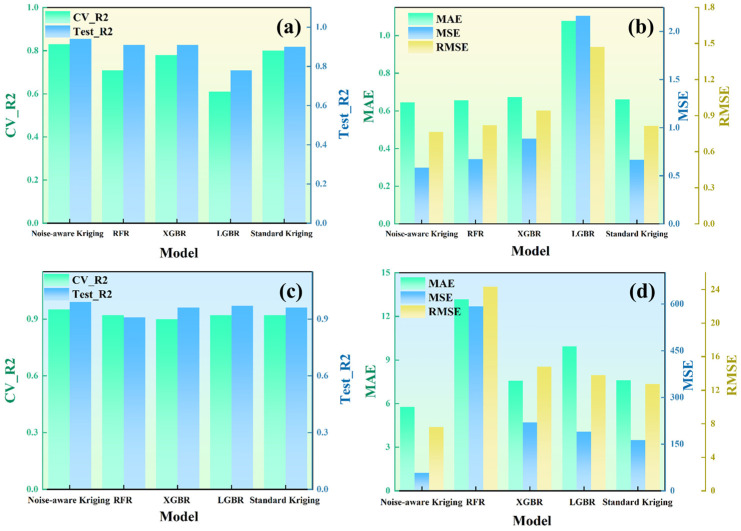
Test-set performance of the machine learning models. (**a**,**b**) Predictive performance of different machine learning models for Δ*H* prediction. (**a**) Five-fold cross-validated coefficient of determination (*CV_R*^2^) and test-set coefficient of determination (*Test_R*^2^). (**b**) Test-set error metrics, including mean absolute error (*MAE*), mean squared error (*MSE*), and root mean squared error (*RMSE*). (**c**,**d**) Predictive performance of different machine learning models for *M_s_* prediction. (**c**) *CV_R*^2^ and Test_*R*^2^. (**d**) Test-set error metrics, including *MAE*, *MSE*, and *RMSE*.

Figure 3a displays the Δ*H* prediction performance of the noise-aware Kriging model at its optimal noise parameter. For a fair comparison, Bayesian hyperparameter optimization was used to tune the key hyperparameters of the baseline models, so that each baseline model was evaluated under near-optimal settings. The predictive results for Δ*H* are shown in Figure 3b,d. The four plotted models capture the main trend in the Δ*H* dataset. However, the noise-ignoring deterministic baseline models (RFR, XGBR, and LGBR) exhibit noticeable scatter and some outlier points on the test set. The standard Kriging model is not shown in Figure 3 for visual clarity but is included in the quantitative comparison in Figure 4. In contrast, the test-set points for the noise-aware Kriging model are much more tightly clustered along the 45° line, indicating that the noise-aware Kriging provides the most accurate Δ*H* predictions.

The comparison between the predicted and measured *M_s_* values is presented in Figure 3e–h. The RFR, XGBR, and LGBR models again show various degrees of scatter and deviation from the diagonal on the test set, whereas the noise-aware Kriging model’s points are more closely aligned to the 45° line. This indicates that the noise-aware Kriging model provides more accurate *M_s_* predictions and exhibits stronger generalization capability to unseen data.

Because training performance is of limited significance for model selection, greater emphasis was placed on generalization to unseen data. Model performance was evaluated using the coefficient of determination (*R*^2^) and error metrics (*MAE*, *MSE*, *RMSE*). Figure 4a,b show the Δ*H* prediction results for the five models. The noise-aware Kriging model attains the highest *R*^2^ on the test set (*Test_R*^2^ = 0.94) and in five-fold cross-validation (*CV_R*^2^ = 0.83), along with the lowest prediction errors (*MAE* = 0.65, *MSE* = 0.58, *RMSE* = 0.76). Notably, its *MSE* is reduced by 13.2% relative to the best deterministic baseline model and by 12.2% relative to the standard Kriging model. Figure 4c,d illustrate the model performance for *M_s_* predictions. All models achieve *Test_R*^2^ and *CV_R*^2^ values above 0.90, indicating that each model captures the overall trend well. However, the noise-aware Kriging model yields significantly lower prediction errors than the deterministic baseline model and standard Kriging model. Its *MAE*, *MSE*, and *RMSE* are 5.78, 58.02, and 7.62, respectively, with the *MSE* reduced by 69.5% relative to the best deterministic baseline model and by 64.3% relative to the standard Kriging model.

The superior performance of the noise-aware Kriging model can be attributed to its treatment of data noise. Baseline models that ignore noise often misinterpret experimental “deviations/fluctuations” as genuine structure–performance patterns. This misinterpretation skews their learned relationships, causing predictions to deviate from the true functional mapping and often leading to overfitting with unstable generalization. In contrast, the noise-aware Kriging model explicitly incorporates data uncertainty as a “noise variance” term in its covariance structure. By automatically reducing confidence in predictions for regions with high uncertainty, it diminishes the influence of outlier data, suppresses extreme prediction errors, and brings the predicted values closer to the underlying true function.

After obtaining the optimized noise-aware Kriging models, the Shapley Additive Explanations (SHAP) analysis was further performed on the predicted mean to examine the model-level contributions of different descriptors to the predictions of Δ*H* and *M_s_*. As shown in Figure S4a,b, positive SHAP values indicate an increase in the model prediction, whereas negative values indicate a decrease, and the color scale represents the feature value from low to high. For Δ*H* prediction, *HOF* exhibits the largest contribution, followed by *SH* and *BP*. Higher *HOF* values generally contribute positively to the predicted Δ*H*, whereas higher *SH* values tend to contribute negatively. *TC*, *RATE*, *CR*, and *TE* show comparatively smaller contributions. For *M_s_* prediction, *EA* shows the largest contribution, followed by *AN*, *BP*, *HOF*, and *CR*. Higher *EA* values generally increase the predicted *M_s_*, whereas higher *AN* values tend to decrease it. *SH*, *RATE*, and *TE* exhibit relatively minor contributions.

To further assess the predictive reliability of the optimized noise-aware Kriging model for unseen Cu-Al-Ni compositions, four literature-reported Cu-Al-Ni compositions that were not included in the original training dataset were used as an independent external validation set. Their Δ*H* values were predicted using the optimized Δ*H* model and compared with the corresponding literature-reported experimental values. As shown in Table 1, the predicted values agree well with the reported values, with relative errors ranging from −4.86% to −0.29%, indicating the good predictive reliability of the optimized noise-aware Kriging model for unseen Cu-Al-Ni compositions.

In summary, the noise-aware Kriging model outperforms the baseline RFR, XGBR, LGBR, and standard Kriging models in both predictive accuracy and generalization ability (Figure 4). In addition, the independent validation results in Table 1 further confirm its prediction reliability for unseen Cu-Al-Ni compositions. Therefore, the optimized noise-aware Kriging model was selected for subsequent Δ*H* and *M_s_* prediction and composition screening across the Cu-Al-Ni compositional space.

### 3.5. Composition Screening and Experimental Validation

**Figure 5:**
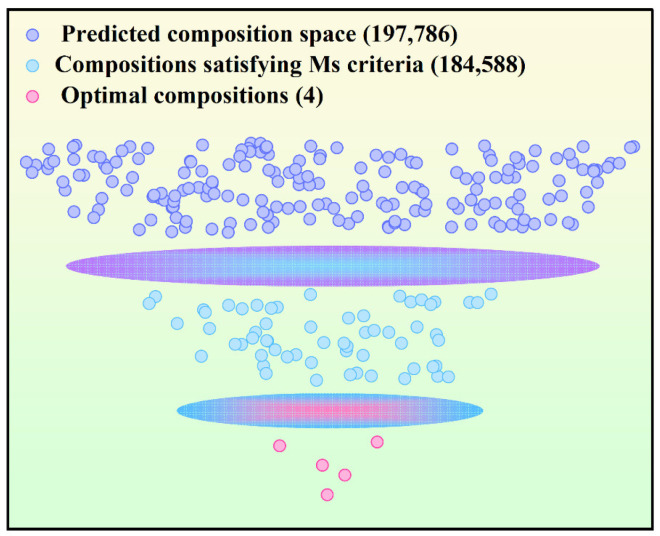
Composition-screening process guided by the noise-aware machine learning strategy.

As shown in Figure 5, to identify the best-performing Cu-Al-Ni compositions for experimentation, the trained noise-aware Kriging model was first used to predict Δ*H* and *M_s_* for all 197,786 potential Cu-Al-Ni compositions in the virtual compositional space. Compositions with predicted *M_s_* values in the target range of 100–200 °C were then retained to ensure transformation within the desired operating temperature window, yielding 184,588 candidate compositions. Finally, the retained candidates were ranked by jointly considering the predicted Δ*H* values and the *SKO* acquisition scores. Because the virtual screening was performed in atomic percent, the selected compositions were converted to weight percent for alloy preparation. Four top-ranked compositions were selected for experimental validation: Cu_84_Al_13_Ni_3_, C_u83.9_Al_13.1_Ni_3_, Cu_83.8_Al_13.2_Ni_3_, and Cu_83.7_Al_13.2_Ni_3.1_ (all compositions are given in wt.%; details are provided in Table S5). These four candidate alloys were then synthesized and characterized via DSC.

**Figure 6:**
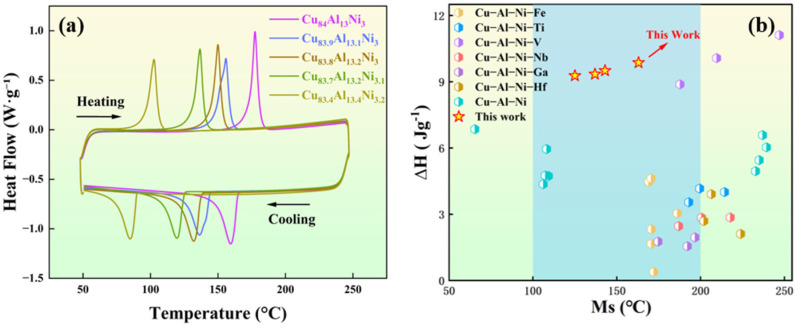
(**a**) DSC curves of the experimental samples. (**b**) Data distribution of Cu–Al–Ni–X alloys, where X denotes an alloying element.

After synthesis, DSC was used to characterize the martensitic transformation behavior of the four selected Cu-Al-Ni samples. Figure 6a shows the DSC curves of the experimental samples. During heating and cooling, each alloy exhibits pronounced endothermic/exothermic peaks, indicating that all four alloys undergo a reversible martensitic transformation within the tested temperature range, accompanied by significant latent-heat absorption and release. This confirms the potential of Cu-Al-Ni solid–solid phase-change materials for thermal management of electronic devices. The transformation temperatures (*M_s_*, *M_f_*, *A_s_*, *A_f_*) were determined from the DSC curves by the intercept method, and the ∆*H* was calculated by integrating the endothermic peak on the heating curve. The specific measured values for each sample are given in Table 2. The ∆*H* values of the four experimental alloys range from 9.27 to 9.86 J/g, and their *M_s_* values range from 125 °C to 163 °C. Among them, the Cu_84_Al_13_Ni_3_ (wt.%) alloy exhibits the highest ∆*H*, reaching 9.86 J/g, with an *M_s_* of approximately 163 °C.

As shown in Figure 6b, within the target temperature range of 100–200 °C, the Δ*H* values of the four Cu-Al-Ni samples developed in this work are substantially higher than those of other literature-reported Cu-Al-Ni-X alloys, where X denotes an alloying element. This result demonstrates that the design goal of developing high-Δ*H* Cu-Al-Ni SMAs within the 100–200 °C window was successfully achieved and supports the effectiveness of the noise-aware machine learning strategy for materials design under limited-data and high-noise conditions.

**Figure 7:**
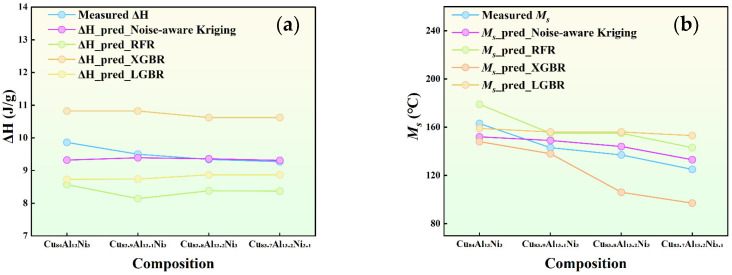
(**a**) Comparison between measured and predicted Δ*H* for the experimental alloys. (**b**) Comparison between measured and predicted *M_s_* for the experimental alloys.

To further verify the accuracy of the model predictions, four machine learning models were used to predict the properties of the experimentally validated compositions. The measured Δ*H* values were then compared with the model predictions, as shown in Figure 7a. It can be seen that the predictions from the noise-aware Kriging model are the closest to the experimental values, showing the best agreement. In contrast, the noise-ignoring models (RFR, XGBR, LGBR) exhibit larger deviations, tending to either over-predict or under-predict overall. Similarly, as shown in Figure 7b for *M_s_*, the RFR, XGBR, and LGBR models produce significant errors for some samples, whereas the noise-aware Kriging model’s predictions are in much closer agreement with the measured values.

To quantitatively assess predictive accuracy, the relative error of each model prediction with respect to the corresponding experimental value was calculated using Equation (S11). The results listed in Table S6 show that, for Δ*H* predictions, the maximum relative error among the noise-ignoring models (RFR, XGBR, LGBR) is 14.54%, whereas the noise-aware Kriging model’s maximum relative error is only 5.43%, corresponding to a reduction of approximately 9 percentage points. For *M_s_* predictions, the results listed in Table S7 show that the maximum relative error reaches 22.74% for the noise-ignoring models, whereas the noise-aware Kriging model confines the maximum error to 6.75%, corresponding to a reduction of approximately 16 percentage points.

Together, these results demonstrate that explicit noise representation enables the noise-aware Kriging model to achieve higher prediction accuracy and reliability than the noise-ignoring baseline models. The reduced extreme prediction errors further support its use as a robust basis for composition screening and experimental validation.

### 3.6. Thermal Performance

**Figure 8:**
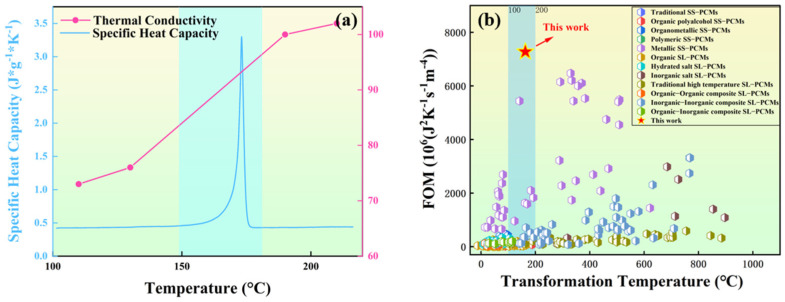
(**a**) Temperature dependence of specific heat capacity (*C_p_*) and thermal conductivity (k) of the Cu_84_Al_13_Ni_3_ (wt.%) alloy. The shaded region indicates the temperature range of the reverse martensite-to-austenite transformation. (**b**) Figure of merit (*FOM*) as a function of phase transition temperature for different types of PCMs.

In the transient thermal management of high-power electronic devices, specific heat capacity (*C_p_*) and thermal conductivity (*k*) determine a material’s thermal energy storage capacity and heat transfer rate, respectively. The Cu_84_Al_13_Ni_3_ (wt.%) alloy, which exhibited the highest ∆*H* among the validated samples, was selected for detailed thermal-performance evaluation and structural characterization. The temperature-dependent *C_p_* and *k* of this alloy are shown in Figure 8, with the shaded region denoting the temperature range over which the reverse (austenitic) transformation occurs. Outside the transformation region, the alloy’s *C_p_* remains in the range of approximately 0.40–0.45 J·g^−1^·K^−1^ and shows very little change with increasing temperature. When the temperature enters the austenitic reverse transformation interval, *C_p_* exhibits a sharp spike (peaking up to 3.3 J·g^−1^·K^−1^). This anomalous increase is caused by the release/absorption of latent heat in the form of “apparent heat capacity” during the phase transformation, and is not a purely intrinsic material property. Therefore, this *C_p_* peak should not be used for performance comparisons across the transformation range or for calculating thermal conductivity [32,33,34].

Using the measured temperature-dependent *α*(*T*), *C_p_* (*T*), and density *ρ*, the thermal conductivity of the Cu_84_Al_13_Ni_3_ alloy was calculated according to *k* = *C_p_*·*α*·*ρ*. Since the measured *C_p_* within the transformation interval contains latent heat, the corresponding *k* in this region should be regarded as an apparent thermal response rather than the intrinsic thermal conductivity. Therefore, quantitative comparison of thermal conductivity in this work is mainly based on the single-phase regions. The results are shown in Figure 8a. In the low-temperature martensitic phase, the alloy shows high thermal conductivity (73–76 W·m^−1^·K^−1^), which gradually increases with increasing temperature. Upon entering the reverse transformation region, *k* rises even more rapidly, and in the high-temperature austenite phase, it reaches a stable value of around 100–102 W·m^−1^·K^−1^. Even in the low-temperature martensitic state, the thermal conductivity of Cu_84_Al_13_Ni_3_ alloy is substantially higher than those of many conventional solid–solid or solid–liquid PCMs listed in Table S8. This high thermal conductivity is favorable for rapid heat transfer from the heat source to the phase-change material, thereby improving the transient thermal response.

High-power electronic devices can generate extremely high heat flux in very short times, so in addition to a high latent-heat capacity, a phase-change cooling material must also exhibit efficient heat transfer to suppress peak temperatures and improve thermal response speed [35,36,37]. For this reason, a figure of merit (*FOM*) was introduced as an application-oriented indicator to evaluate the combined latent-heat storage capacity and heat-transfer capability of the alloys for transient thermal-management applications. To evaluate the thermal performance of the proposed alloys, the *FOM* is defined as *FOM* = *k*·*ρ*·*L* [38,39], where *k* is the thermal conductivity, *ρ* is the density, and *L* is the latent heat per unit mass. The unit of *FOM* is J^2^·K^−1^·s^−1^·m^−4^. Here, *L* corresponds to the measured Δ*H*. A higher *FOM* indicates that a material can absorb/release more heat in a smaller volume at a faster rate, which is especially important for device miniaturization and for managing transient thermal spikes. A comparison with representative phase-change materials, including Cu-Al-Ni alloys, NiTi-based SMAs and other reported solid–solid and solid–liquid PCMs, is summarized in Table S8 and plotted in Figure 8b as *FOM* versus transformation temperature. The Cu_84_Al_13_Ni_3_ (wt.%) alloy developed in this work exhibits an FOM of 7272 × 10^6^ J^2^ K^−1^ s^−1^ m^−4^, which is more than 12-fold higher than the highest value reported for commercial PCM products ((18–584) × 10^6^ J^2^ K^−1^ s^−1^ m^−4^) [40,41]. Within the representative phase-change materials surveyed in Table S8, this alloy exhibits the highest *FOM* in the 100–200 °C phase-change temperature range. The high *FOM* of the Cu-Al-Ni solid–solid PCM arises from the combined contribution of high density, high thermal conductivity, and high Δ*H*. This combination suggests that Cu-Al-Ni SMAs are promising candidates for transient thermal management applications in high-power electronic devices.

**Figure 9:**
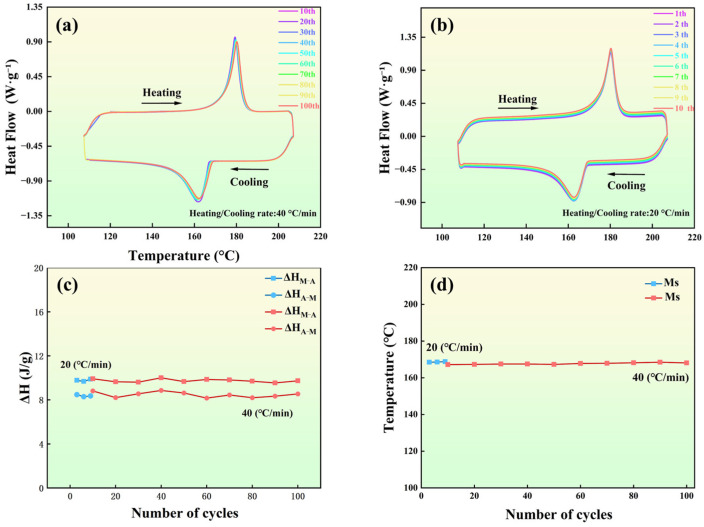
Thermal cycling stability of the Cu_84_Al_13_Ni_3_ (wt.%) alloy under different heating/cooling rates. (**a**) 100 thermal cycles at 40 °C/min; (**b**) 10 thermal cycles at 20 °C/min; (**c**) variation in ΔH with cycle number; (**d**) variation in *M_s_* with cycle number.

Thermal cycling stability is a crucial prerequisite for the long-term application of phase-change materials in high-power electronics [42,43]. To assess performance retention during repeated phase-change cycling, 100 consecutive thermal cycles were conducted on the Cu_84_Al_13_Ni_3_ (wt.%) alloy at a heating/cooling rate of 40 °C/min. As shown in Figure 9a, the positions of the endothermic and exothermic peaks remained nearly unchanged with increasing cycle number, and no obvious shift was observed in either Δ*H* or *M_s_*. These results indicate that the alloy maintains stable phase-transformation behavior even under a relatively high heating/cooling rate, demonstrating good thermal-cycling stability over 100 cycles. Considering that practical devices may experience different heating/cooling rates, 10 supplementary cycles were further performed at a lower rate of 20 °C/min. As shown in Figure 9b, the Δ*H* and *M_s_* also remained essentially unchanged under this slower cycling condition, further confirming the robust cycling stability of the alloy.

To quantitatively evaluate cycling stability, the evolution of thermal properties with cycle number was extracted, as shown in Figure 9c,d. At both heating/cooling rates of 20 °C/min and 40 °C/min, the reverse transformation enthalpy Δ*H*_M→A_ remains in a narrow range of 9.54–10.0 J/g, and the forward transformation enthalpy Δ*H*_A→M_ stays around 8.16–8.86 J/g. Overall, the Δ*H* values exhibit only random fluctuations with cycle number and no monotonic degradation trend. *M_s_* shows almost no change throughout cycling. Even after 100 cycles at 40 °C/min, the Δ*H* decreased by merely 0.158 J/g and *M_s_* shifted by only 0.9 °C. These results indicate that the Cu_84_Al_13_Ni_3_ alloy possesses promising thermal cycling stability and shows good potential for transient thermal-management applications in high-power electronics involving repeated thermal charging and discharging.

### 3.7. Structural Characterization

**Figure 10:**
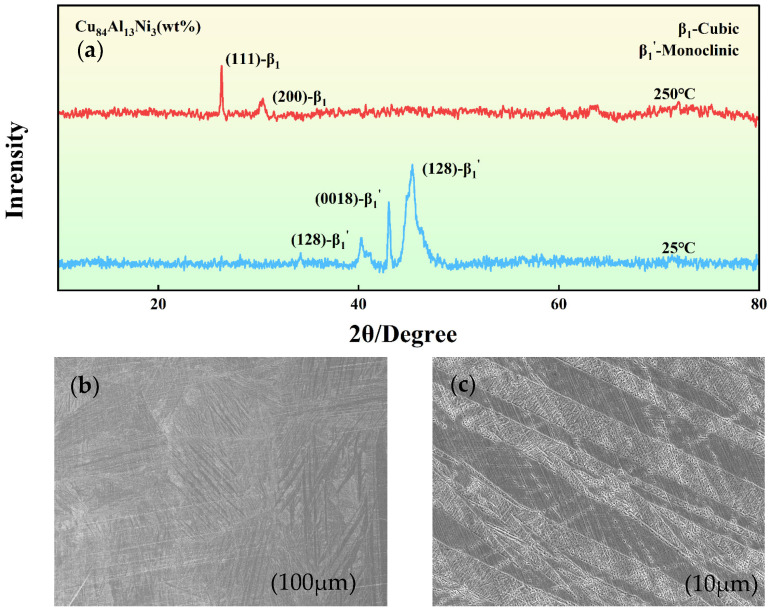
(**a**) Variable-temperature XRD patterns of the Cu_84_Al_13_Ni_3_ (wt.%) alloy. (**b**,**c**) Scanning electron micrographs of the Cu_84_Al_13_Ni_3_ (wt.%) alloy after solution quenching: (**b**) 100 μm scale bar; (**c**) 10 μm scale bar.

Temperature-dependent XRD was carried out from 25 °C to 250 °C to examine the crystal structure evolution during the alloy’s phase transformation (Figure 10a). At room temperature, the main diffraction peaks correspond to the (0018) and (128) planes, which are characteristic of the monoclinic 18R martensite phase. As the temperature is increased to 250 °C, these martensitic peaks disappear and are replaced by the characteristic peaks of the austenite phase, indexed to the (111) and (200) planes, while no additional impurity peaks are detected. This result indicates that the alloy undergoes a martensite-to-austenite phase transformation within the tested temperature range. This structural evolution supports the solid–solid phase transformation basis of latent-heat storage in the Cu-Al-Ni alloy.

To investigate the microstructural morphology of the Cu_84_Al_13_Ni_3_ (wt.%) alloy, the solution-treated and quenched sample was characterized by SEM, as shown in Figure 10b,c. At lower magnification (Figure 10b, scale bar 100 μm), the interior of the alloy is seen to consist of bundles of martensitic laths with different orientations intersecting each other. The lath interfaces are sharp and the laths are relatively narrow, presenting a typical lamellar martensite morphology. At higher magnification (Figure 10c, scale bar 10 μm), each martensite lath is found to contain parallel banded substructures, and fine needle-like martensite is interspersed between the laths. This indicates a multi-scale, multi-variant martensitic microstructure. No second-phase particles or other precipitates are observed, which is consistent with the XRD result that no secondary phases were detected at room temperature. This suggests that the room-temperature microstructure after solution quenching is predominantly martensitic. These structural observations are consistent with the reversible solid–solid phase transformation of the alloy and support its latent-heat storage function.

## 4. Conclusions

This work applied a noise-aware machine learning strategy with dual constraints on *M_s_* and Δ*H* to develop high-Δ*H* Cu-Al-Ni alloys for transient thermal-management applications. By minimizing prediction error, the optimal noise level was determined and incorporated into the noise-aware Kriging model and the SKO acquisition function. Relative to the best deterministic baseline and the standard Kriging model, respectively, the noise-aware Kriging model reduced the *MSE* by 13.2% and 12.2% for Δ*H* prediction and by 69.5% and 64.3% for *M_s_* prediction, indicating that the explicit incorporation of observational noise effectively improved the predictive accuracy of the model. The relative errors on the independent external Cu-Al-Ni validation set ranged from −4.86% to −0.29%, supporting the good generalization capability of the model trained on the Cu-Al-based dataset for unseen Cu-Al-Ni compositions. Guided by this strategy, four Cu-Al-Ni alloys were synthesized and experimentally validated, exhibiting *M_s_* values ranging from 125 to 163 °C and Δ*H* values ranging from 9.27 to 9.86 J/g. Their Δ*H* values all exceeded the previously reported maximum for Cu-Al-Ni alloys within the 100–200 °C range, demonstrating the effectiveness of the noise-aware machine learning strategy for high-Δ*H* alloy screening. The best-performing Cu_84_Al_13_Ni_3_ (wt.%) alloy exhibited *M*_s_ of 163 °C, Δ*H* of 9.86 J/g, *k* of 102 W·m^−1^·K^−1^, and *FOM* of 7272 × 10^6^ J^2^ K^−1^ s^−1^ m^−4^. This *FOM* exceeds the previous highest Cu-Al-Ni *FOM* by 33.75% and represents the highest value among the surveyed PCMs within the 100–200 °C range. After 100 thermal cycles, this alloy showed only a 0.158 J/g decrease in Δ*H* and a 0.9 °C shift in *M*_s_, confirming good thermal-cycling stability. These results demonstrate that the noise-aware machine learning strategy can improve predictive accuracy and candidate-selection reliability for materials design by explicitly incorporating observational noise under small-sample and noisy experimental data conditions, while also demonstrating the potential of Cu-Al-Ni SMAs for transient thermal-management applications in high-power electronic devices.

## Figures and Tables

**Table 1:** Comparison between measured and predicted Δ*H* for independent Cu-Al-Ni validation samples.

Composition	Measured Δ*H*	Predicted Δ*H*	Relative Error
[at.%]	[J/g]	[J/g]	[%]
Cu_69.38_Al_26.97_Ni_3.65_	5.34	5.30	−0.75
Cu_69.46_Al_26.89_Ni_3.65_	5.76	5.48	−4.86
Cu_69.55_Al_26.8_Ni_3.65_	6.85	6.67	−2.63
Cu_69.63_Al_26.72_Ni_3.65_	6.90	6.88	−0.29

**Table 2:** Differential scanning calorimetry (DSC) results for the experimental samples.

Composition [wt.%]	*A*_s_	*A*_f_	*M*_f_	*M*_s_	Δ*H*
	[°C]	[°C]	[°C]	[°C]	[J/g]
Cu_84_ Al_13_ Ni_3_	173	181	149	163	9.86
Cu_83.9_ Al_13.1_ Ni_3_	144	157	123	143	9.5
Cu_83.8_ Al_13.2_ Ni_3_	144	154	121	137	9.34
Cu_83.7_ Al_13.2_Ni_3.1_	127	137	102	125	9.27

## Data Availability

The original contributions presented in this study are included in the article/Supplementary Material. Further inquiries can be directed to the corresponding authors.

## Supplementary Materials

The following supporting information can be downloaded at https://www.mdpi.com/article/10.3390/ma19132802/s1, Figure S1: Data distributions of Cu-based shape-memory alloys. (a) Distribution of latent heat (Δ*H*); (b) distribution of martensitic start temperature (*M*_s_); (c) joint distribution of Δ*H* and *M*_s_; Figure S2: Feature selection for latent heat prediction model; Figure S3: Feature selection for martensitic transformation temperatures; Figure S4: SHAP interpretability analysis of the optimized noise-aware Kriging models. (a) SHAP summary plot for Δ*H* prediction. (b) SHAP summary plot for *M_s_* prediction. Positive SHAP values indicate positive contributions to the model prediction, whereas negative values indicate negative contributions. The color scale represents the feature value from low to high; Table S1: Phase transformation latent heat of Cu-based shape memory alloys collected from the literature; Table S2: Martensitic transformation start temperature (*M_s_*) of Cu-based shape memory alloys collected from the literature; Table S3: The input feature parameters of the Cu-Al-Ni SMA Δ*H* and *M*_s_ dataset; Table S4: Final features for two property prediction models of shape memory alloys; Table S5: Chemical compositions of four Cu-Al-Ni candidate alloys recommended by the noise-aware active learning strategy; Table S6: Relative errors between predicted and experimentally measured Δ*H* values; Table S7: Relative error between the predicted and measured *M_s_* values; Table S8: Thermophysical properties of typical SS-PCMs and SL-PCMs.
