# Supplementary material for: Noise-Aware Machine Learning Accelerates Development of High-Latent-Heat Cu-Al-Ni Shape Memory Alloys for Thermal Management
Source: Materials (Basel). 2026 Jul 1;19(13):2802. doi: 10.3390/ma19132802 (PMC13362812; doi:10.3390/ma19132802)
Supplement: Supplementary file 1 [file materials-19-02802-s001.zip › materials-4208801-supplementary.pdf]

Supplementary Information

Noise-Aware Machine Learning Accelerates Development of High Latent Heat Cu-Al-Ni Shape Memory Alloys for Thermal Management

Noise-aware Kriging model. Assuming that repeated measurements under identical composition and processing conditions follow a Gaussian distribution, an observation can be expressed as $\overline{y}_i = y_i + \varepsilon_i\,, \varepsilon_i \sim \mathcal{N}(0, \tau^2)$, where $\overline{y}_i$ is the literature observation, y_i is the true value, ε_i is the noise term, and τ^2 is the noise variance. The covariance matrix between training data points accordingly includes an additional noise term, $K_{noisy} = K + \Delta$, where $\Delta$is a diagonal matrix containing only the measurement-noise variances. Within the GPR framework, the predictive mean μ and predictive uncertainty s^2 for an arbitrary query point x can be written as

$$\mu = f(x)^*\xi + K(x^*, x)^*(K + \Delta)^{-1}(\overline{y} - f(x^*)\xi) \qquad (S1)$$

$$s^2 = s_{SK}^2 + (f(x)^* - K(x^*, x)^*(K + \Delta)^{-1}f(x^*))^*(f(x^*)^*(K + \Delta)^{-1}f(x^*))^{-1} (f(x)^* - K(x^*, x)^*(K + \Delta)^{-1}f(x^*)) \qquad (S2)$$

where $\tilde{y} = (\tilde{y}_1, \ldots, \tilde{y}_p)^\top$ denotes the noisy observations, p is the number of labeled training points x^*, $f(x)$ is the vector of trend-function values at x, $f(x^*)$ is the experimental matrix, and ξ is the corresponding coefficient vector

Determination of the noise level and hyperparameter selection. Because the intrinsic noise level of the Cu-Al-Ni dataset is unknown, we calibrated the assumed measurement noise by scaling it with the response range:

$$\tau^2 = \left[\Phi\left(y^{max} - y^{min}\right)\right]^2, \phi(0,1) \qquad (S3)$$

where $y^{max}$and y^{min} are the maximum and minimum values of the target property in the dataset, and Φ is a prefactor controlling the noise magnitude. We treated Φ as a hyperparameter and searched it over a broad range. For each Φ, model performance was evaluated via leave-one-out cross-validation (*LOO-CV*) using both the mean squared error (*MSE*) and the continuous ranked probability score (CRPS), and the Φ that minimized both metrics was selected to train the final model.

$$MSE = \frac{\sum_{i=1}^{n}(\bar{y_i} - \mu)^2}{n} \qquad (S4)$$

$$CRPS = s\left(\frac{1}{\sqrt{\pi}} - 2\varphi\left(\frac{\overline{y}_i - \mu}{s}\right) - \frac{\overline{y}_i - \mu}{s}\left(2\Psi\left(\frac{\overline{y}_i - \mu}{s}\right) - 1\right)\right) \qquad (S5)$$

where n is the number of validation samples, and φ and Ψ are the standard normal probability density function and cumulative distribution function, respectively.

Noise-enhanced acquisition in sequential Kriging optimization. To guide experimental selection, a noise-enhanced acquisition function was adopted within sequential Kriging optimization (*SKO*):

$$v_{SKO} = \left(1 - \frac{\tau}{\sqrt{\tau^2 + s^2}}\right) sG\left(\frac{u - u^{**}}{s}\right) \quad \text{(S6)}$$

where $u^{**} = u_{(argmax[u - \lambda s])}$, λ is the "risk-avoid" parameter. u and s denote the predicted mean and standard deviation provided by the Kriging model, respectively, whereas τ^2 represents the variance of the observation distribution for each sample, corresponding to data uncertainty.

Model performance evaluation. The dataset was randomly split into a training set and a test set at an 8:2 ratio. Metrics reported on the test set were used to quantify the model's extrapolative performance on previously unseen samples. In addition, five-fold cross-validation was performed and the cross-validated coefficient of determination (CV_R²) was calculated to assess the stability and robustness of the model under different data partitions.

Let y_i denote the literature-reported (observed) value, $\hat{y}_i$ denote the predicted value, n denote the number of samples in the test set (or the current validation fold), and $\bar{y} = \frac{1}{n}\sum_{i=1}^{n} y_i$ denote the mean of the observations.

Coefficient of determination (R²) (Test_R² and CV_R² are computed using the same definition):

$$R^2 = 1 - \frac{\sum_{i=1}^{n}(y_i - \hat{y}_i)^2}{\sum_{i=1}^{n}(y_i - \bar{y})^2} \quad \text{(S7)}$$

R^2 measures the fraction of the variance in the target variable explained by the model; values closer to 1 indicate better agreement between predictions and observations.

Mean absolute error (MAE), mean squared error (MSE), and root mean squared error (RMSE) are defined as

$$MAE = \frac{1}{n}\sum_{i=1}^{n} |y_i - \hat{y}_i| \quad \text{(S8)}$$

$$MSE = \frac{1}{n}\sum_{i=1}^{n} (y_i - \hat{y}_i)^2 \quad \text{(S9)}$$

$$RMSE = \sqrt{\frac{1}{n}\sum_{i=1}^{n} (y_i - \hat{y}_i)^2} \quad \text{(S10)}$$

MAE, MSE, and RMSE quantify prediction errors; smaller values indicate higher predictive accuracy.

For the Kriging model, the point prediction is taken as the predictive mean $\mu(x)$. To jointly evaluate the predictive distribution and its consistency with observations, the continuous ranked probability score (CRPS) was used to assess uncertainty; a smaller CRPS indicates better agreement between the predictive distribution and the observed value.

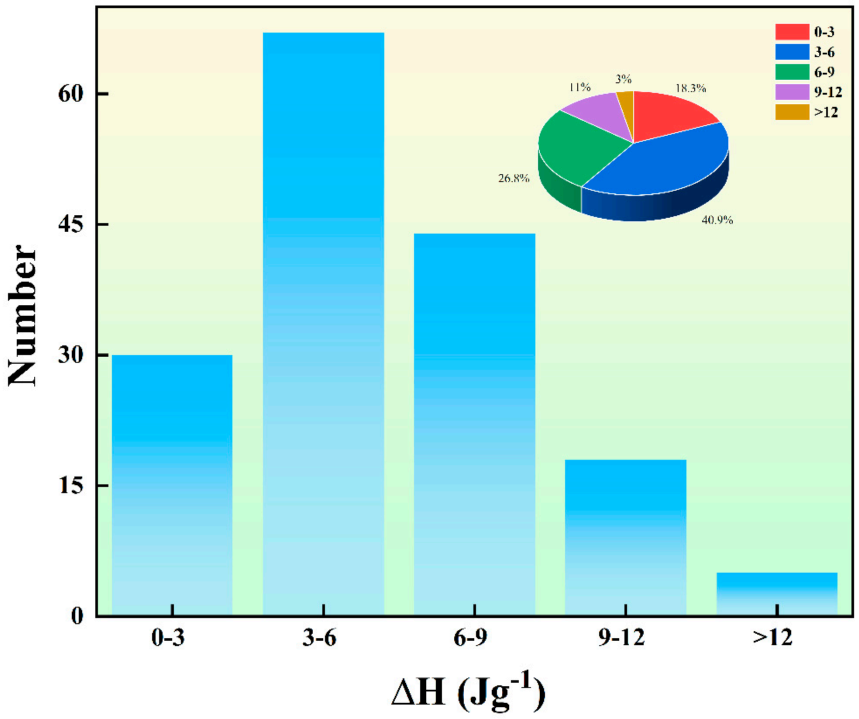

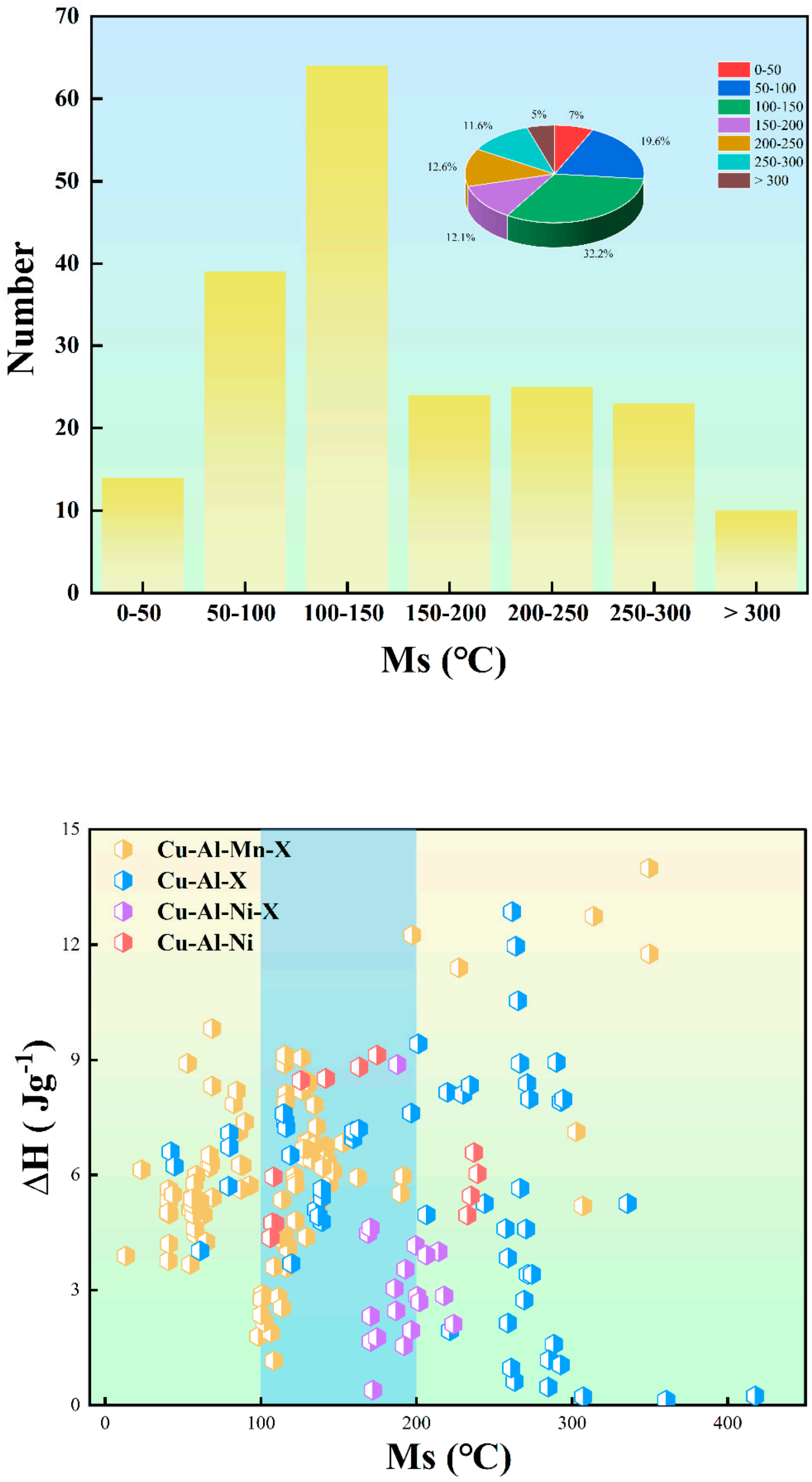

Figure S1. Data distributions of Cu-based shape-memory alloys. (a) Distribution of latent heat (ΔH); (b) distribution of martensitic start temperature (M_s); (c) joint distribution of ΔH and M_s.

Table S1. Phase transformation latent heat of Cu-based shape memory alloys collected from the literature

No.	Alloy (at.%)	$\Delta H_{M\rightarrow A}$ (J/g)	Ref.
1	$Cu_{71.38}Al_{24.08}Fe_{2.23}Co_{1.34}Ti_{0.97}$	0.11	[1]
2	$Cu_{73.64}Al_{22.6}Mn_{3.18}Fe_{0.57}$	4.38	[2]
3	$Cu_{79.99}Al_{15.54}Ni_{3.56}Fe_{0.91}$	1.66	[3]
4	$Cu_{79.99}Al_{15.54}Ni_{3.56}Fe_{0.91}$	4.47	[3]
5	$Cu_{77.32}Al_{18.97}Ni_{3.48}Nb_{0.23}$	2.85	[4]
6	$Cu_{70.5}Al_{24.57}Mn_{3.25}Mg_{1.69}$	6.65	[5]
7	$Cu_{77.32}Al_{18.97}Ni_{3.48}Nb_{0.23}$	2.46	[4]
8	$Cu_{79.88}Al_{12.59}Ni_{4.17}Ga_{3.36}$	1.55	[4]
9	$Cu_{72.65}Al_{23.01}Mn_{4.34}$	4.04	[6]
10	$Cu_{77.62}Al_{17.88}Ni_{3.74}V_{0.76}$	8.88	[4]
11	$Cu_{73.1}Al_{23.03}Mn_{3.16}Fe_{0.7}$	3.58	[2]
12	$Cu_{72.19}Al_{22.78}Fe_{2.59}Mn_{2.44}$	6.51	[7]
13	$Cu_{85.14}Al_{11.19}Mn_{3.67}$	1.16	[8]
14	$Cu_{68.9}Al_{27.18}Ni_{3.91}$	4.36	[9]
15	$Cu_{73.04}Al_{21.52}Fe_{3.49}Mn_{1.95}$	5.63	[7]
16	$Cu_{74.15}Al_{20.28}Mn_{5.57}$	11.09	[10]
17	$Cu_{70}Al_{25}Mn_{4}Zr_{1}$	7.13	[11]
18	$Cu_{70.53}Al_{24.95}Mn_{3.45}Fe_{1.05}$	2.89	[2]
19	$Cu_{70}Al_{25}Mn_{4}Zr_{1}$	5.19	[11]
20	$Cu_{91.42}Al_{8.25}Te_{0.33}$	9.42	[8]
21	$Cu_{67.94}Al_{26.49}Mn_{4.4}Mg_{1.17}$	5.99	[5]
22	$Cu_{73.23}Al_{21.93}Fe_{3.36}Mn_{1.48}$	5.7	[12]
23	$Cu_{70}Al_{25}Mn_{4}V_{1}$	5.24	[11]
24	$Cu_{77.62}Al_{17.88}Ni_{3.74}V_{0.76}$	10.07	[4]
25	$Cu_{74.78}Al_{22.22}Fe_{3}$	12.86	[7]
26	$Cu_{73.64}Al_{22.6}Mn_{3.18}Fe_{0.57}$	5.28	[2]
27	$Cu_{78.89}Al_{15.67}Ni_{4.1}Ti_{1.33}$	3.54	[4]
28	$Cu_{73.04}Al_{21.52}Fe_{3.49}Mn_{1.95}$	4.92	[7]
29	$Cu_{91.42}Al_{8.25}Te_{0.33}$	8.34	[8]
30	$Cu_{72.68}Al_{24.07}Ti_{1.85}Co_{1.4}$	4.6	[1]
31	$Cu_{74.82}Al_{22.12}Mn_{3.06}$	9.13	[13]
32	$Cu_{85.14}Al_{11.19}Mn_{3.67}$	1.89	[8]
33	$Cu_{73.04}Al_{21.52}Fe_{3.49}Mn_{1.95}$	4.78	[7]
34	$Cu_{68.9}Al_{27.18}Ni_{3.91}$	4.73	[9]
35	$Cu_{78.25}Al_{17.2}Ni_{2.81}Fe_{1.74}$	4.62	[3]
36	$Cu_{74.78}Al_{22.22}Fe_{3}$	8.91	[7]
37	$Cu_{69.99}Al_{26.05}Ni_{3.96}$	5.45	[9]

38	$Cu_{70}Al_{25}Mn_4Ga_1$	5.31	[11]
39	$Cu_{72.68}Al_{24.07}Ti_{1.85}Co_{1.4}$	0.97	[1]
40	$Cu_{69.21}Al_{26.08}Mn_{2.85}Mg_{1.86}$	8.36	[13]
41	$Cu_{70}Al_{25}Mn_4V_1$	5.77	[11]
42	$Cu_{70.53}Al_{24.95}Mn_{3.45}Fe_{1.05}$	2.54	[2]
43	$Cu_{72.68}Al_{24.07}Ti_{1.85}Co_{1.4}$	5.25	[1]
44	$Cu_{71.38}Al_{24.08}Fe_{2.23}Co_{1.34}Ti_{0.97}$	0.13	[1]
45	$Cu_{70}Al_{25}Mn_4Ga_1$	4.25	[11]
46	$Cu_{85.14}Al_{11.19}Mn_{3.67}$	2.35	[8]
47	$Cu_{73.8}Al_{20.96}Mn_{3.87}Fe_{1.37}$	7.65	[5]
48	$Cu_{78.89}Al_{15.67}Ni_{4.1}Ti_{1.33}$	4	[4]
49	$Cu_{72.68}Al_{24.07}Ti_{1.85}Co_{1.4}$	3.84	[1]
50	$Cu_{73.1}Al_{23.03}Mn_{3.16}Fe_{0.7}$	5.97	[2]
51	$Cu_{79.99}Al_{15.54}Ni_{3.56}Fe_{0.91}$	0.39	[3]
52	$Cu_{89.67}Al_{9.65}Te_{0.68}$	5.65	[8]
53	$Cu_{73.8}Al_{20.96}Mn_{3.87}Fe_{1.37}$	8.2	[5]
54	$Cu_{69.99}Al_{26.05}Ni_{3.96}$	6.58	[9]
55	$Cu_{90.54}Al_{8.25}Sn_{1.21}$	1.18	[8]
56	$Cu_{70}Al_{25}Mn_4Cr_1$	5.96	[11]
57	$Cu_{70.69}Al_{24.39}Mn_{3.92}Fe_1$	5.25	[5]
58	$Cu_{82.2}Al_{13.15}Ni_{4.24}Fe_{0.41}$	12.09	[3]
59	$Cu_{70.58}Al_{26.31}Ni_{3.12}$	8.47	[14]
60	$Cu_{70.53}Al_{24.95}Mn_{3.45}Fe_{1.05}$	3.6	[2]
61	$Cu_{71.08}Al_{25.8}Ni_{3.12}$	9.13	[14]
62	$Cu_{70.69}Al_{24.39}Mn_{3.92}Fe_1$	5.26	[5]
63	$Cu_{91.42}Al_{8.25}Te_{0.33}$	8.11	[8]
64	$Cu_{72.19}Al_{22.78}Fe_{2.59}Mn_{2.44}$	3.69	[7]
65	$Cu_{70.69}Al_{24.39}Mn_{3.92}Fe_1$	7.38	[5]
66	$Cu_{70.81}Al_{22.79}Fe_{5.42}Mn_{0.98}$	10.18	[7]
67	$Cu_{90.54}Al_{8.25}Sn_{1.21}$	1.05	[8]
68	$Cu_{69.99}Al_{26.05}Ni_{3.96}$	4.95	[9]
69	$Cu_{78.89}Al_{15.67}Ni_{4.1}Ti_{1.33}$	4.16	[4]
70	$Cu_{87.59}Al_{12.1}Hf_{0.31}$	3.41	[8]
71	$Cu_{82.2}Al_{13.15}Ni_{4.24}Fe_{0.41}$	10.39	[3]
72	$Cu_{70}Al_{25}Mn_4Nb_1$	5.1	[11]
73	$Cu_{67.45}Al_{27.96}Mn_{4.6}$	4.19	[10]
74	$Cu_{73.64}Al_{22.6}Mn_{3.18}Fe_{0.57}$	5.64	[2]
75	$Cu_{73.23}Al_{21.93}Fe_{3.36}Mn_{1.48}$	7.09	[12]
76	$Cu_{85.14}Al_{11.19}Mn_{3.67}$	2.76	[8]
77	$Cu_{75.57}Al_{23.31}V_{1.22}$	8.94	[15]
78	$Cu_{73.8}Al_{20.96}Mn_{3.87}Fe_{1.37}$	5.79	[5]

79	$Cu_{73.04}Al_{21.52}Fe_{3.49}Mn_{1.95}$	5.41	[7]
80	$Cu_{71.38}Al_{24.08}Fe_{2.23}Co_{1.34}Ti_{0.97}$	0.22	[1]
81	$Cu_{70.81}Al_{22.79}Fe_{5.42}Mn_{0.98}$	10.46	[7]
82	$Cu_{70}Al_{25}Mn_{4}Ga_{1}$	5.42	[11]
83	$Cu_{72.65}Al_{23.01}Mn_{4.34}$	3.19	[6]
84	$Cu_{73.1}Al_{23.03}Mn_{3.16}Fe_{0.7}$	5.73	[2]
85	$Cu_{73.23}Al_{21.93}Fe_{3.36}Mn_{1.48}$	4.02	[12]
86	$Cu_{82.2}Al_{13.15}Ni_{4.24}Fe_{0.41}$	11.74	[3]
87	$Cu_{70.81}Al_{22.79}Fe_{5.42}Mn_{0.98}$	11.75	[7]
88	$Cu_{71.38}Al_{24.08}Fe_{2.23}Co_{1.34}Ti_{0.97}$	0.24	[1]
89	$Cu_{73.64}Al_{22.6}Mn_{3.18}Fe_{0.57}$	6.19	[2]
90	$Cu_{90.54}Al_{8.25}Sn_{1.21}$	0.62	[8]
91	$Cu_{72.65}Al_{23.01}Mn_{4.34}$	6.51	[6]
92	$Cu_{82.2}Al_{13.15}Ni_{4.24}Fe_{0.41}$	12.45	[3]
93	$Cu_{74.82}Al_{22.12}Mn_{3.06}$	8.89	[13]
94	$Cu_{73.8}Al_{20.96}Mn_{3.87}Fe_{1.37}$	8.9	[5]
95	$Cu_{72.19}Al_{22.78}Fe_{2.59}Mn_{2.44}$	7.23	[7]
96	$Cu_{70}Al_{25}Mn_{4}Nb_{1}$	5.06	[11]
97	$Cu_{78.25}Al_{17.2}Ni_{2.81}Fe_{1.74}$	3.04	[3]
98	$Cu_{70}Al_{25}Mn_{4}Nb_{1}$	5.09	[11]
99	$Cu_{69.21}Al_{26.08}Mn_{2.85}Mg_{1.86}$	8.2	[13]
100	$Cu_{73.04}Al_{21.52}Fe_{3.49}Mn_{1.95}$	5.09	[7]
101	$Cu_{73.1}Al_{23.03}Mn_{3.16}Fe_{0.7}$	4.8	[2]
102	$Cu_{89.67}Al_{9.65}Te_{0.68}$	4.59	[8]
103	$Cu_{71.89}Al_{22.53}Ti_{5.31}Mg_{0.26}$	5.25	[16]
104	$Cu_{70.5}Al_{24.57}Mn_{3.25}Mg_{1.69}$	6.21	[5]
105	$Cu_{73.64}Al_{22.6}Mn_{3.18}Fe_{0.57}$	6.09	[2]
106	$Cu_{70.69}Al_{24.39}Mn_{3.92}Fe_{1}$	6.16	[5]
107	$Cu_{69.21}Al_{26.08}Mn_{2.85}Mg_{1.86}$	9.05	[13]
108	$Cu_{73.23}Al_{21.93}Fe_{3.36}Mn_{1.48}$	9.76	[12]
109	$Cu_{68.9}Al_{27.18}Ni_{3.91}$	5.95	[9]
110	$Cu_{70}Al_{25}Mn_{4}Zr_{1}$	13.99	[11]
111	$Cu_{70}Al_{25}Mn_{4}Nb_{1}$	4.96	[11]
112	$Cu_{70}Al_{25}Mn_{4}V_{1}$	4.61	[11]
113	$Cu_{74.82}Al_{22.12}Mn_{3.06}$	8.12	[13]
114	$Cu_{70}Al_{25}Mn_{4}V_{1}$	5.38	[11]
115	$Cu_{70}Al_{25}Mn_{4}Ga_{1}$	3.67	[11]
116	$Cu_{70}Al_{25}Mn_{4}Cr_{1}$	12.25	[11]
117	$Cu_{70.53}Al_{24.95}Mn_{3.45}Fe_{1.05}$	2.82	[2]
118	$Cu_{70}Al_{25}Mn_{4}Nb_{1}$	4.92	[11]
119	$Cu_{67.94}Al_{26.49}Mn_{4.4}Mg_{1.17}$	5.07	[5]

120	$Cu_{79.88}Al_{12.59}Ni_{4.17}Ga_{3.36}$	1.95	[4]
121	$Cu_{72.65}Al_{23.01}Mn_{4.34}$	6.86	[6]
122	$Cu_{89.67}Al_{9.65}Te_{0.68}$	3.41	[8]
123	$Cu_{69.99}Al_{26.05}Ni_{3.96}$	6.03	[9]
124	$Cu_{70.81}Al_{22.79}Fe_{5.42}Mn_{0.98}$	7.62	[7]
125	$Cu_{70.5}Al_{24.57}Mn_{3.25}Mg_{1.69}$	5.49	[5]
126	$Cu_{79.25}Al_{18.13}Ni_{2.49}Hf_{0.13}$	2.69	[4]
127	$Cu_{74.78}Al_{22.22}Fe_{3}$	8.39	[7]
128	$Cu_{74.78}Al_{22.22}Fe_{3}$	10.54	[7]
129	$Cu_{70}Al_{25}Mn_{4}Zr_{1}$	11.76	[11]
130	$Cu_{87.59}Al_{12.1}Hf_{0.31}$	2.74	[8]
131	$Cu_{70.38}Al_{26.56}Mn_{3.06}$	7.86	[10]
132	$Cu_{67.94}Al_{26.49}Mn_{4.4}Mg_{1.17}$	5.78	[5]
133	$Cu_{60}Al_{24}Hf_{9.5}Mn_{6}Zr_{0.5}$	6.23	[17]
134	$Cu_{73.23}Al_{21.93}Fe_{3.36}Mn_{1.48}$	6.73	[12]
135	$Cu_{70}Al_{25}Mn_{4}Cr_{1}$	5.93	[11]
136	$Cu_{72.19}Al_{22.78}Fe_{2.59}Mn_{2.44}$	7.61	[7]
137	$Cu_{70.91}Al_{25.97}Ni_{3.12}$	8.82	[14]
138	$Cu_{85.14}Al_{11.19}Mn_{3.67}$	2.16	[8]
139	$Cu_{73.1}Al_{23.03}Mn_{3.16}Fe_{0.7}$	5.81	[2]
140	$Cu_{68.23}Al_{26}Mn_{4.33}Ni_{1.44}$	6.13	[18]
141	$Cu_{89.67}Al_{9.65}Te_{0.68}$	2.14	[8]
142	$Cu_{77.62}Al_{17.88}Ni_{3.74}V_{0.76}$	11.11	[4]
143	$Cu_{79.88}Al_{12.59}Ni_{4.17}Ga_{3.36}$	1.76	[4]
144	$Cu_{69}Al_{24}Mn_{6}Zr_{1}$	4.2	[17]
145	$Cu_{79.25}Al_{18.13}Ni_{2.49}Hf_{0.13}$	3.91	[4]
146	$Cu_{82.2}Al_{13.15}Ni_{4.24}Fe_{0.41}$	11.8	[3]
147	$Cu_{70}Al_{25}Mn_{4}V_{1}$	4.97	[11]
148	$Cu_{75.57}Al_{23.31}V_{1.22}$	7.98	[15]
149	$Cu_{90.54}Al_{8.25}Sn_{1.21}$	0.47	[8]
150	$Cu_{72.19}Al_{22.78}Fe_{2.59}Mn_{2.44}$	7.4	[7]
151	$Cu_{70.53}Al_{24.95}Mn_{3.45}Fe_{1.05}$	1.79	[2]
152	$Cu_{60}Al_{24}Hf_{10}Mn_{6}$	6.6	[17]
153	$Cu_{68.9}Al_{27.18}Ni_{3.91}$	4.75	[9]
154	$Cu_{69.21}Al_{26.08}Mn_{2.85}Mg_{1.86}$	8.49	[13]
155	$Cu_{75.57}Al_{23.31}V_{1.22}$	7.92	[15]
156	$Cu_{77.32}Al_{18.97}Ni_{3.48}Nb_{0.23}$	2.84	[4]
157	$Cu_{70}Al_{25}Mn_{4}Ga_{1}$	4.45	[11]
158	$Cu_{72.5}Al_{24.7}Mn_{2.81}$	6.83	[10]
159	$Cu_{70}Al_{25}Mn_{4}Cr_{1}$	11.4	[11]
160	$Cu_{91.42}Al_{8.25}Te_{0.33}$	8.16	[8]

161	$Cu_{70.74}Al_{26.14}Ni_{3.12}$	8.52	[14]
162	$Cu_{74.78}Al_{22.22}Fe_{3}$	11.96	[7]
163	$Cu_{79.99}Al_{15.54}Ni_{3.56}Fe_{0.91}$	2.32	[3]
164	$Cu_{74.82}Al_{22.12}Mn_{3.06}$	7.88	[13]

Table S2. Martensitic transformation start temperature (M_s) of Cu-based shape memory alloys collected from the literature

No.	Alloy (at.%)	M_s(°C)	Ref.
1	$Cu_{73.04}Al_{21.52}Fe_{3.49}Mn_{1.95}$	85.98	[6]
2	$Cu_{79.25}Al_{18.13}Ni_{2.49}Hf_{0.13}$	108.35	[7]
3	$Cu_{70}Al_{25}Mn_{4}Ga_{1}$	107.40	[1]
4	$Cu_{72.68}Al_{24.07}Ti_{1.85}Co_{1.4}$	108.65	[2]
5	$Cu_{70.81}Al_{22.79}Fe_{5.42}Mn_{0.98}$	349.77	[11]
6	$Cu_{70.74}Al_{26.14}Ni_{3.12}$	187.74	[4]
97	$Cu_{73.63}Al_{21.44}Mn_{4.38}Ti_{0.55}$	234.37	[8]
8	$Cu_{91.42}Al_{8.25}Te_{0.33}$	257.37	[1]
9	$Cu_{70}Al_{25}Mn_{4}Ga_{1}$	307.26	[1]
10	$Cu_{73.64}Al_{22.6}Mn_{3.18}Fe_{0.57}$	128.25	[19]
11	$Cu_{90.54}Al_{8.25}Sn_{1.21}$	115.62	[7]
12	$Cu_{70}Al_{25}Mn_{4}Cr_{1}$	206.41	[4]
13	$Cu_{70.43}Al_{26.65}Ni_{2.93}$	110.00	[14]
14	$Cu_{74.78}Al_{22.22}Fe_{3}$	82.66	[10]
15	$Cu_{70}Al_{25}Mn_{4}Cr_{1}$	113.58	[2]
16	$Cu_{91.42}Al_{8.25}Te_{0.33}$	186.93	[4]
17	$Cu_{74.55}Al_{21.38}Mn_{3.77}$	197.30	[11]
18	$Cu_{72.19}Al_{22.78}Fe_{2.59}Mn_{2.44}$	313.82	[11]
19	$Cu_{69.7}Al_{26.17}Mn_{2.5}Mg_{1.64}$	194.72	[7]
20	$Cu_{75.76}Al_{20.48}Mn_{2.03}Cr_{1.73}$	55.35	[11]
21	$Cu_{69.76}Al_{26.07}Mn_{2.85}Mg_{1.32}$	289.99	[20]
22	$Cu_{69.99}Al_{26.05}Ni_{3.96}$	190.01	[11]
23	$Cu_{69.38}Al_{26.97}Ni_{3.65}$	1.30	[14]
24	$Cu_{69.21}Al_{26.08}Mn_{2.85}Mg_{1.86}$	67.28	[6]
25	$Cu_{68.9}Al_{27.18}Ni_{3.91}$	214.71	[3]
26	$Cu_{70}Al_{25}Mn_{4}Nb_{1}$	270.43	[8]
27	$Cu_{68.9}Al_{27.18}Ni_{3.91}$	57.35	[11]
28	$Cu_{70}Al_{25}Mn_{4}Zr_{1}$	221.56	[7]
29	$Cu_{69.99}Al_{26.05}Ni_{3.96}$	58.20	[11]
30	$Cu_{73.23}Al_{21.93}Fe_{3.36}Mn_{1.48}$	285.19	[8]

31	$Cu_{71.08}Al_{25.8}Ni_{3.12}$	135.61	[7]
32	$Cu_{72.19}Al_{22.78}Fe_{2.59}Mn_{2.44}$	54.84	[11]
33	$Cu_{70.53}Al_{24.95}Mn_{3.45}Fe_{1.05}$	360.54	[1]
34	$Cu_{73.04}Al_{21.52}Fe_{3.49}Mn_{1.95}$	192.69	[7]
35	$Cu_{72.65}Al_{23.01}Mn_{4.34}$	98.44	[2]
36	$Cu_{72.65}Al_{23.01}Mn_{4.34}$	146.82	[2]
37	$Cu_{73.63}Al_{21.44}Mn_{4.38}Ti_{0.55}$	201.15	[8]
38	$Cu_{87.59}Al_{12.1}Hf_{0.31}$	306.90	[11]
39	$Cu_{67.94}Al_{26.49}Mn_{4.4}Mg_{1.17}$	229.95	[8]
40	$Cu_{70.53}Al_{24.95}Mn_{3.45}Fe_{1.05}$	57.70	[11]
41	$Cu_{78.89}Al_{15.67}Ni_{4.1}Ti_{1.33}$	191.66	[7]
42	$Cu_{75.57}Al_{23.31}V_{1.22}$	232.80	[9]
43	$Cu_{74.71}Al_{22.01}Mn_{3.07}Cr_{0.21}$	137.73	[2]
44	$Cu_{79.88}Al_{12.59}Ni_{4.17}Ga_{3.36}$	214.27	[4]
45	$Cu_{72.19}Al_{22.78}Fe_{2.59}Mn_{2.44}$	129.23	[17]
46	$Cu_{74.71}Al_{22.01}Mn_{3.07}Cr_{0.21}$	266.55	[8]
47	$Cu_{79.25}Al_{18.13}Ni_{2.49}Hf_{0.13}$	106.59	[8]
48	$Cu_{71.89}Al_{22.53}Ti_{5.31}Mg_{0.26}$	192.10	[4]
49	$Cu_{70}Al_{25}Mn_{4}V_{1}$	140.81	[19]
50	$Cu_{73.64}Al_{22.6}Mn_{3.18}Fe_{0.57}$	223.87	[4]
51	$Cu_{70.53}Al_{24.95}Mn_{3.45}Fe_{1.05}$	119.72	[7]
52	$Cu_{74.82}Al_{22.12}Mn_{3.06}$	272.51	[8]
53	$Cu_{74.82}Al_{22.12}Mn_{3.06}$	292.82	[15]
54	$Cu_{70.53}Al_{24.95}Mn_{3.45}Fe_{1.05}$	68.78	[6]
55	$Cu_{69.54}Al_{25.84}Mn_{3.42}Mg_{1.21}$	13.30	[19]
56	$Cu_{77.62}Al_{17.88}Ni_{3.74}V_{0.76}$	130.44	[13]
57	$Cu_{70.81}Al_{22.79}Fe_{5.42}Mn_{0.98}$	84.62	[5]
58	$Cu_{73.81}Al_{21.64}Mn_{3.57}Cr_{0.99}$	63.30	[11]
59	$Cu_{69.99}Al_{26.05}Ni_{3.96}$	126.39	[13]
60	$Cu_{75.57}Al_{23.31}V_{1.22}$	116.35	[3]
61	$Cu_{70}Al_{25}Mn_{4}V_{1}$	44.60	[17]
62	$Cu_{70.81}Al_{22.79}Fe_{5.42}Mn_{0.98}$	58.28	[11]
63	$Cu_{73.1}Al_{23.03}Mn_{3.16}Fe_{0.7}$	114.72	[7]
64	$Cu_{72.5}Al_{24.7}Mn_{2.81}$	79.34	[12]
65	$Cu_{74.78}Al_{22.22}Fe_{3}$	52.94	[2]
66	$Cu_{91.42}Al_{8.25}Te_{0.33}$	203.99	[3]
67	$Cu_{69}Al_{24}Mn_{6}Zr_{1}$	92.78	[6]
68	$Cu_{87.59}Al_{12.1}Hf_{0.31}$	139.36	[7]

69	$Cu_{70.81}Al_{22.79}Fe_{5.42}Mn_{0.98}$	139.14	[7]
70	$Cu_{70}Al_{25}Mn_4V_1$	141.73	[19]
71	$Cu_{73.81}Al_{21.64}Mn_{3.57}Cr_{0.99}$	200.54	[4]
72	$Cu_{74.78}Al_{22.22}Fe_3$	239.30	[9]
73	$Cu_{72.19}Al_{22.78}Fe_{2.59}Mn_{2.44}$	335.63	[13]
74	$Cu_{70}Al_{25}Mn_4Cr_1$	170.54	[3]
75	$Cu_{79.99}Al_{15.54}Ni_{3.56}Fe_{0.91}$	88.23	[6]
76	$Cu_{82.2}Al_{13.15}Ni_{4.24}Fe_{0.41}$	112.38	[19]
77	$Cu_{71.3}Al_{22.9}Mn_{4.87}Ni_{0.9}$	217.71	[4]
78	$Cu_{73.64}Al_{22.6}Mn_{3.18}Fe_{0.57}$	116.54	[2]
79	$Cu_{90.54}Al_{8.25}Sn_{1.21}$	213.87	[18]
80	$Cu_{73.63}Al_{21.44}Mn_{4.38}Ti_{0.55}$	246.98	[4]
81	$Cu_{72.19}Al_{22.78}Fe_{2.59}Mn_{2.44}$	58.20	[11]
82	$Cu_{60}Al_{24}Hf_{10}Mn_6$	67.84	[6]
83	$Cu_{75.76}Al_{20.48}Mn_{2.03}Cr_{1.73}$	130.51	[21]
84	$Cu_{70}Al_{25}Mn_4Zr_1$	186.11	[3]
85	$Cu_{70}Al_{25}Mn_4V_1$	109.30	[1]
86	$Cu_{73.23}Al_{21.93}Fe_{3.36}Mn_{1.48}$	121.96	[2]
87	$Cu_{71.3}Al_{22.9}Mn_{4.87}Ni_{0.9}$	132.18	[5]
88	$Cu_{72.68}Al_{24.07}Ti_{1.85}Co_{1.4}$	170.62	[3]
89	$Cu_{69.55}Al_{26.80}Ni_{3.65}$	65.40	[14]
90	$Cu_{70}Al_{25}Mn_4V_1$	129.82	[19]
91	$Cu_{70}Al_{25}Mn_4Ga_1$	79.97	[12]
92	$Cu_{70}Al_{25}Mn_4Nb_1$	170.43	[3]
93	$Cu_{74.71}Al_{22.01}Mn_{3.07}Cr_{0.21}$	196.64	[7]
94	$Cu_{73.23}Al_{21.93}Fe_{3.36}Mn_{1.48}$	260.83	[1]
95	$Cu_{70.74}Al_{26.14}Ni_{3.12}$	141.80	[14]
96	$Cu_{70}Al_{25}Mn_4Cr_1$	263.90	[7]
97	$Cu_{70}Al_{25}Mn_4Zr_1$	227.27	[11]
98	$Cu_{69.21}Al_{26.08}Mn_{2.85}Mg_{1.86}$	69.09	[10]
99	$Cu_{77.32}Al_{18.97}Ni_{3.48}Nb_{0.23}$	79.90	[12]
100	$Cu_{70.69}Al_{24.39}Mn_{3.92}Fe_1$	135.02	[19]
101	$Cu_{79.99}Al_{15.54}Ni_{3.56}Fe_{0.91}$	269.33	[8]
102	$Cu_{70.58}Al_{26.31}Ni_{3.12}$	125.70	[14]
103	$Cu_{70}Al_{25}Mn_4Zr_1$	271.17	[22]
104	$Cu_{70}Al_{25}Mn_4Nb_1$	292.67	[8]
105	$Cu_{70}Al_{25}Mn_4Nb_1$	43.71	[5]
106	$Cu_{70}Al_{24}Mn_6$	349.50	[11]

107	$Cu_{70.91}Al_{25.97}Ni_{3.12}$	163.30	[14]
108	$Cu_{70}Al_{25}Mn_{4}Zr_{1}$	122.23	[2]
109	$Cu_{78.25}Al_{17.2}Ni_{2.81}Fe_{1.74}$	274.27	[8]
110	$Cu_{70.81}Al_{22.79}Fe_{5.42}Mn_{0.98}$	56.05	[11]
111	$Cu_{73.04}Al_{21.52}Fe_{3.49}Mn_{1.95}$	117.44	[19]
112	$Cu_{70.91}Al_{25.97}Ni_{3.12}$	127.31	[13]
113	$Cu_{90.54}Al_{8.25}Sn_{1.21}$	143.22	[19]
114	$Cu_{60}Al_{24}Hf_{9.5}Mn_{6}Zr_{0.5}$	86.70	[6]
115	$Cu_{68.9}Al_{27.18}Ni_{3.91}$	132.62	[19]
116	$Cu_{74.82}Al_{22.12}Mn_{3.06}$	115.71	[19]
117	$Cu_{85.14}Al_{11.19}Mn_{3.67}$	235.00	[9]
118	$Cu_{79.25}Al_{18.13}Ni_{2.49}Hf_{0.13}$	199.48	[4]
119	$Cu_{73.8}Al_{20.96}Mn_{3.87}Fe_{1.37}$	108.20	[1]
120	$Cu_{71.38}Al_{24.08}Fe_{2.23}Co_{1.34}Ti_{0.97}$	261.57	[7]
121	$Cu_{89.67}Al_{9.65}Te_{0.68}$	41.10	[17]
122	$Cu_{73.63}Al_{21.44}Mn_{4.38}Ti_{0.55}$	41.23	[6]
123	$Cu_{73.04}Al_{21.52}Fe_{3.49}Mn_{1.95}$	113.71	[19]
124	$Cu_{69.21}Al_{26.08}Mn_{2.85}Mg_{1.86}$	284.70	[8]
125	$Cu_{79.88}Al_{12.59}Ni_{4.17}Ga_{3.36}$	68.77	[6]
126	$Cu_{82.2}Al_{13.15}Ni_{4.24}Fe_{0.41}$	122.33	[2]
127	$Cu_{71.08}Al_{25.8}Ni_{3.12}$	174.70	[14]
128	$Cu_{71.89}Al_{22.53}Ti_{5.31}Mg_{0.26}$	169.01	[3]
129	$Cu_{73.81}Al_{21.64}Mn_{3.57}Cr_{0.99}$	219.95	[8]
130	$Cu_{74.78}Al_{22.22}Fe_{3}$	40.71	[6]
131	$Cu_{71.38}Al_{24.08}Fe_{2.23}Co_{1.34}Ti_{0.97}$	61.29	[11]
132	$Cu_{71.89}Al_{22.53}Ti_{5.31}Mg_{0.26}$	23.48	[18]
133	$Cu_{74.82}Al_{22.12}Mn_{3.06}$	206.04	[3]
134	$Cu_{68.08}Al_{26.11}Mn_{4.27}Ni_{1.54}$	116.31	[7]
135	$Cu_{73.64}Al_{22.6}Mn_{3.18}Fe_{0.57}$	172.01	[3]
136	$Cu_{85.14}Al_{11.19}Mn_{3.67}$	111.38	[2]
137	$Cu_{73.04}Al_{21.52}Fe_{3.49}Mn_{1.95}$	134.55	[21]
138	$Cu_{75.57}Al_{23.31}V_{1.22}$	162.38	[11]
139	$Cu_{78.89}Al_{15.67}Ni_{4.1}Ti_{1.33}$	196.67	[4]
140	$Cu_{70.5}Al_{24.57}Mn_{3.25}Mg_{1.69}$	192.94	[8]
141	$Cu_{82.2}Al_{13.15}Ni_{4.24}Fe_{0.41}$	207.73	[3]
142	$Cu_{71.3}Al_{22.9}Mn_{4.87}Ni_{0.9}$	206.45	[8]
143	$Cu_{82.2}Al_{13.15}Ni_{4.24}Fe_{0.41}$	129.23	[19]
144	$Cu_{70.53}Al_{24.95}Mn_{3.45}Fe_{1.05}$	139.01	[2]

145	$Cu_{89.67}Al_{9.65}Te_{0.68}$	89.87	[5]
146	$Cu_{85.14}Al_{11.19}Mn_{3.67}$	106.20	[9]
147	$Cu_{77.62}Al_{17.88}Ni_{3.74}V_{0.76}$	115.25	[13]
148	$Cu_{73.23}Al_{21.93}Fe_{3.36}Mn_{1.48}$	54.65	[15]
149	$Cu_{73.63}Al_{21.44}Mn_{4.38}Ti_{0.55}$	263.09	[8]
150	$Cu_{79.99}Al_{15.54}Ni_{3.56}Fe_{0.91}$	115.55	[13]
151	$Cu_{89.67}Al_{9.65}Te_{0.68}$	266.43	[7]
152	$Cu_{72.65}Al_{23.01}Mn_{4.34}$	144.21	[19]
153	$Cu_{73.1}Al_{23.03}Mn_{3.16}Fe_{0.7}$	56.78	[11]
154	$Cu_{91.42}Al_{8.25}Te_{0.33}$	100.00	[8]
155	$Cu_{73.81}Al_{21.64}Mn_{3.57}Cr_{0.99}$	294.36	[15]
156	$Cu_{73.1}Al_{23.03}Mn_{3.16}Fe_{0.7}$	191.34	[11]
157	$Cu_{85.14}Al_{11.19}Mn_{3.67}$	101.13	[2]
158	$Cu_{73.23}Al_{21.93}Fe_{3.36}Mn_{1.48}$	258.92	[1]
159	$Cu_{68.9}Al_{27.18}Ni_{3.91}$	56.05	[11]
160	$Cu_{85.14}Al_{11.19}Mn_{3.67}$	243.88	[4]
161	$Cu_{74.71}Al_{22.01}Mn_{3.07}Cr_{0.21}$	79.60	[12]
162	$Cu_{70}Al_{25}Mn_{4}Ga_{1}$	174.64	[4]
163	$Cu_{90.54}Al_{8.25}Sn_{1.21}$	258.83	[8]
164	$Cu_{71.3}Al_{22.9}Mn_{4.87}Ni_{0.9}$	66.69	[6]
165	$Cu_{69.46}Al_{26.89}Ni_{3.65}$	35.60	[14]
166	$Cu_{77.32}Al_{18.97}Ni_{3.48}Nb_{0.23}$	127.47	[19]
167	$Cu_{82.2}Al_{13.15}Ni_{4.24}Fe_{0.41}$	40.82	[6]
168	$Cu_{87.59}Al_{12.1}Hf_{0.31}$	60.99	[11]
169	$Cu_{72.68}Al_{24.07}Ti_{1.85}Co_{1.4}$	139.89	[2]
170	$Cu_{75.76}Al_{20.48}Mn_{2.03}Cr_{1.73}$	42.20	[17]
171	$Cu_{74.55}Al_{21.38}Mn_{3.77}$	126.54	[13]
172	$Cu_{79.99}Al_{15.54}Ni_{3.56}Fe_{0.91}$	139.22	[7]
173	$Cu_{74.55}Al_{21.38}Mn_{3.77}$	102.38	[8]
174	$Cu_{74.78}Al_{22.22}Fe_{3}$	55.93	[11]
175	$Cu_{69.99}Al_{26.05}Ni_{3.96}$	271.68	[8]
176	$Cu_{75.76}Al_{20.48}Mn_{2.03}Cr_{1.73}$	116.10	[10]
177	$Cu_{78.89}Al_{15.67}Ni_{4.1}Ti_{1.33}$	237.00	[9]
178	$Cu_{69.63}Al_{26.72}Ni_{3.65}$	39.50	[14]
179	$Cu_{71.3}Al_{22.9}Mn_{4.87}Ni_{0.9}$	201.95	[4]
180	$Cu_{72.65}Al_{23.01}Mn_{4.34}$	302.79	[11]
181	$Cu_{70}Al_{25}Mn_{4}Ga_{1}$	65.08	[11]
182	$Cu_{70.58}Al_{26.31}Ni_{3.12}$	121.64	[2]

183	$Cu_{72.68}Al_{24.07}Ti_{1.85}Co_{1.4}$	136.03	[12]
184	$Cu_{89.67}Al_{9.65}Te_{0.68}$	119.14	[7]
185	$Cu_{75.57}Al_{23.31}V_{1.22}$	40.48	[6]
186	$Cu_{70.38}Al_{26.56}Mn_{3.06}$	40.89	[6]
187	$Cu_{74.55}Al_{21.38}Mn_{3.77}$	307.23	[16]
188	$Cu_{69.21}Al_{26.08}Mn_{2.85}Mg_{1.86}$	58.58	[5]
189	$Cu_{77.32}Al_{18.97}Ni_{3.48}Nb_{0.23}$	288.24	[15]
190	$Cu_{73.64}Al_{22.6}Mn_{3.18}Fe_{0.57}$	115.95	[13]
191	$Cu_{79.88}Al_{12.59}Ni_{4.17}Ga_{3.36}$	137.17	[7]
192	$Cu_{72.65}Al_{23.01}Mn_{4.34}$	87.23	[6]
193	$Cu_{77.62}Al_{17.88}Ni_{3.74}V_{0.76}$	209.57	[4]
194	$Cu_{73.1}Al_{23.03}Mn_{3.16}Fe_{0.7}$	265.38	[7]
195	$Cu_{73.1}Al_{23.03}Mn_{3.16}Fe_{0.7}$	309.23	[23]
196	$Cu_{78.25}Al_{17.2}Ni_{2.81}Fe_{1.74}$	152.82	[10]
197	$Cu_{72.65}Al_{23.01}Mn_{4.34}$	130.90	[19]
198	$Cu_{91.42}Al_{8.25}Te_{0.33}$	100.60	[8]

Feature Construction and Selection.

We assembled 22 candidate descriptors spanning three categories: (i) intrinsic elemental physical properties (e.g., atomic radius, CR; average valence electron concentration, CVEN); (ii) thermodynamic/thermophysical descriptors relevant to martensitic transformation (e.g., boiling point, BP; enthalpy of vaporization, HOV; thermal expansion coefficient, TE); and (iii) processing parameters (e.g., heating/cooling rate, RATE; solution-treatment temperature, STT). All descriptors and their definitions are summarized in Table S3. Using the full descriptor set can inflate model complexity, increasing computational burden and susceptibility to overfitting. We therefore implemented a hybrid feature-selection workflow that couples random forest (RF) importance ranking with correlation-based redundancy removal, enabling dimensionality reduction while preserving information content.

The workflow comprised three steps:

（i.） RF importance ranking: For each target property (ΔH and M_s), an RF model was trained to quantify and rank the relative importance of the 22 descriptors (bar plots in Figs. S2 and S3).

（ii.） Correlation screening: Pairwise absolute Pearson correlation coefficients ($|r|$) were computed across all descriptors and visualized as heatmaps (Figs. S2 and S3), where darker colors indicate stronger correlation and thus higher redundancy.

（iii.） Redundancy pruning: For any descriptor pair with $|r| > 0.9$, only the descriptor with the higher RF importance was retained.

As a result, seven descriptors (HOV, RATE, BP, TE, SH, CR, and TC) were selected for ΔH prediction, while eight descriptors (BP, TE, SH, CR, EA, HOF, AN, and RATE) were retained for M_s prediction.

Table S3. The input feature parameters of the Cu–Al–Ni SMA ΔH and M_s dataset.

Abbreviation	Description
AN	Atomic Number
AM	Atomic Mass
DE	Density
SH	Specific Heat
TC	Thermal Conductivity
VEN	Valence Electron Concentration
PEN	Electronegativity Pauling
EA	Electron Affinity
CVEN	Average Valence Electron Concentration
EC	Electrical Conductivity
AR	Atomic Radius
CR	Covalent Radius
DOR	Atomic Waber–Crome Pseudopotential Radius
MP	Melting Point
HOV	Heat of Vaporization
HOF	Heat of Fusion
BP	Boiling Point
RATE	Heating/Cooling Rate
STT	Solution Treatment Temperature
DT	Solution Treatment Time
TE	Thermal Expansion
MV	Molar Volume
$\Delta H_{M\rightarrow A}$	Enthalpy of Transformation from Martensite to Austenite

Table S4. Final features for two property prediction models of shape memory alloys.

Objective	Features
High phase-change latent heat (ΔH)	*HOF*, *RATE*, *BP*, *TE*, *SH*, *CR*, *TC*
Martensitic transformation temperatures (M_s)	*BP*, *TE*, *SH*, *CR*, *EA*, *HOF*, *AN*, *RATE*

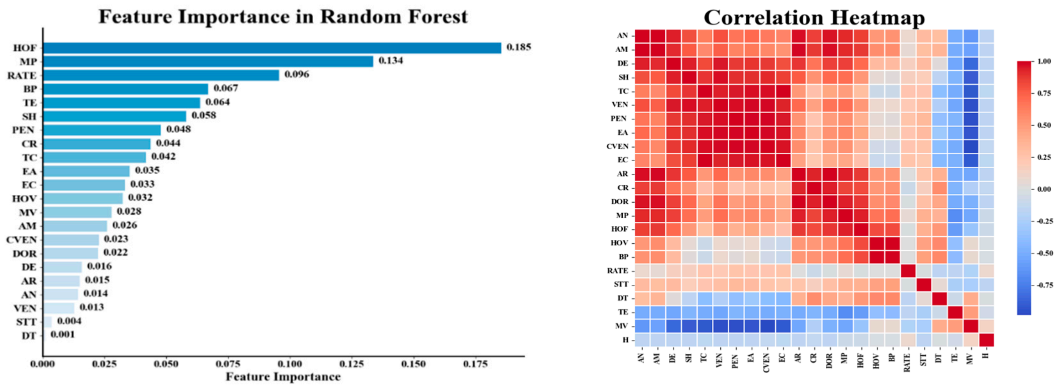

Figure S2. Feature selection for latent heat prediction model.

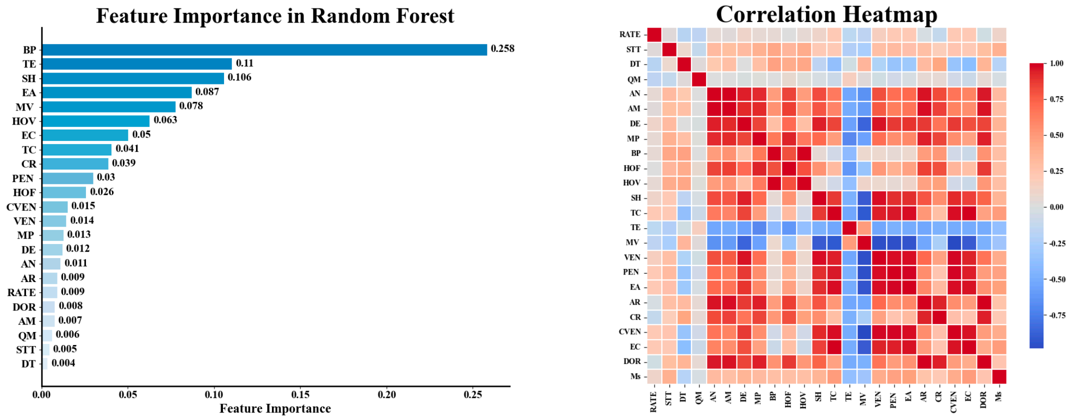

Figure S3. Feature selection for martensitic transformation temperatures.

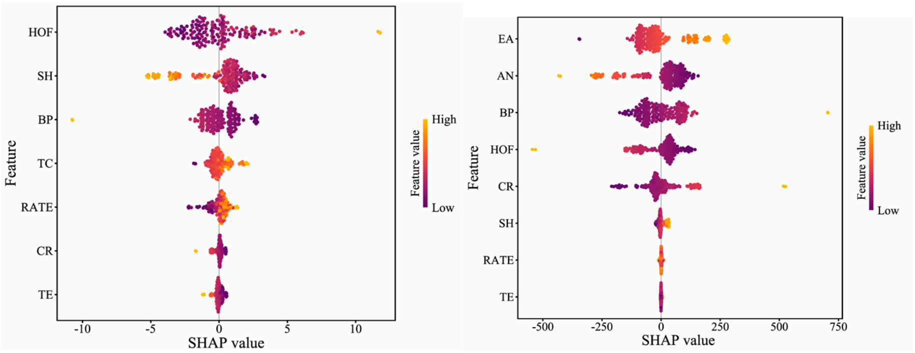

Figure S4. SHAP interpretability analysis of the optimized noise-aware Kriging models. (a) SHAP summary plot for ΔH prediction. (b) SHAP summary plot for M_s prediction. Positive SHAP values indicate positive contributions to the model prediction, whereas negative values indicate negative contributions. The color scale represents the feature value from low to high.

Table S5. Chemical compositions of four Cu-Al-Ni candidate alloys recommended by the noise-aware active learning strategy

Cu (at.%)	Al (at.%)	Ni (at.%)	Cu (wt.%)	Al (wt.%)	Ni (wt.%)	Cu (g)	Al (g)	Ni (g)
71.22	26.06	2.72	84	13	3	8.4000	1.3000	0.3000
71.09	26.19	2.72	83.9	13.1	3	8.3909	1.3125	0.2965
71.01	26.24	2.75	83.8	13.2	3	8.3846	1.3155	0.2999
70.88	26.32	2.8	83.7	13.2	3.1	8.3741	1.3203	0.3055

Assuming that the predicted value of the i-th sample is $\hat{y}_i$and the corresponding measured value is y_i, the relative error (percentage) between the prediction and the measurement is defined as

$$RE_i(\%) = \frac{\hat{y}_i - y_i}{y_i} \times 100\% \quad \text{(S11)}$$

A smaller RE indicates that the predicted value is closer to the measured value.

Table S6. Relative errors between predicted and experimentally measured ΔH values

Alloy(wt%) Model	Cu_{84} Al_{13} Ni_3	$Cu_{83.9}$ $Al_{13.1}$ Ni_3	$Cu_{83.8}$ $Al_{13.2}$ Ni_3	$Cu_{83.8}$ $Al_{13.2}$ Ni_3	Max.RE(%)
Noise-aware Kriging	-5.43%	-1.21%	0.24%	0.46%	5.43%
RFR	-13.05%	-14.32%	-10.31%	-9.75%	-14.32%
XGBR	9.78%	13.86%	13.67%	14.54%	14.54%
LGBR	-11.43%	-7.99%	-4.99%	-4.27%	-11.43%

Table S7. Relative error between the predicted and measured M_s values

Alloy(wt%) Model	Cu_{84} Al_{13} Ni_3	$Cu_{83.9}$ $Al_{13.1}$ Ni_3	$Cu_{83.8}$ $Al_{13.2}$ Ni_3	$Cu_{83.8}$ $Al_{13.2}$ Ni_3	Max.RE(%)
Noise-aware Kriging	-6.75%	4.20%	5.11%	6.40%	6.75%
RFR	9.80%	8.72%	13.48%	14.10%	14.10%
XGBR	-9.28%	-3.42%	-22.74%	-22.07%	22.74%
LGBR	-2.62%	9.22%	14.00%	22.28%	22.28%

Table S8. Thermophysical properties of typical SS-PCMs and SL-PCMs

Materials	Transition temperature (°C)	Latent heat (kJ/kg^{-1})	Density (10^3 kg/m^{-3})	Thermal conductivity (W/m^{-1} K^{-1})	FOM (10^6 J^2 K^{-1} s^{-1} m^{-4})	Ref.
**Traditional SS-PCMs (commercial materials)**						
PlusICE X25	25	110	1.055	0.36	42	[1]
PlusICE X30	30	115	1.05	0.36	44	[1]
PlusICE X40	40	150	1.046	0.36	57	[1]
PlusICE X55	55	115	1.06	0.36	44	[1]
PlusICE X70	70	160	1.085	0.36	63	[1]
PlusICE X80	80	160	1.193	0.36	69	[1]
PlusICE X90	90	170	1.2	0.36	73	[1]
PlusICE X95	95	140	1.215	0.36	61	[1]
PlusICE X120	120	185	1.245	0.36	83	[1]
PlusICE X130	130	315	1.28	0.36	145	[1]
PlusICE X165	165	225	1.304	0.36	105	[1]
PlusICE X180	180	275	1.33	0.36	132	[1]
**Organic polyalcohol SS-PCMs (non-commercial materials)**						
Neopentyl glycol (NPG)	48	131.0	1.06	0.116	16	[2, 3]
Pentaerythritol (PE)	188	303.3	1.39	0.177	75	[2, 3]
Pentaglycerine (PG)	89	193.0	1.22	0.072	17	[2, 3]
**Organometallic SS-PCMs (non-commercial materials)**						
C10Mn	32.8	70.34	1.5	2	211	[4, 6]
C10Cu	33.8	62.57	1.5	2	188	[4, 6]
C12Cu	52.5	70.15	1.5	2	210	[4, 6]
C12Mn	54.1	80.80	1.5	2	242	[4, 6]
C12Co	60.7	92.89	1.5	2	279	[4, 6]
C14Cu	69.2	163.99	1.2	2	394	[4, 7]
C15Cu	72.3	126.42	1.1	2	278	[4, 7]
C16Cu	72.8	79.76	1.5	2	239	[4, 6]
C16Mn	73.1	104.48	1.5	2	313	[4, 6]
C10Co	77.7	74.28	1.5	2	223	[4, 6]
C10Zn	80.1	100.92	1.5	2	303	[4, 6]
C12Zn	88.2	120.23	1.5	2	361	[4, 6]
C16Co	93.4	153.79	1.5	2	461	[4, 6]
C16Zn	99.1	137.52	1.5	2	413	[4, 6]
**Polymeric SS-PCMs (non-commercial materials)**						
S-AA-SE(4:1)	27	34	1.015	0.12	4	[7]
S-AA-SE(2:1)	28	63	0.984	0.13	8	[7]
P(S-SE)	29	74	0.978	0.15	11	[7]
**Metallic SS-PCMs (non-commercial materials)**						

$Ni_{47.73}Ti_{52.27}$ (at.%)	57	13.5	6.239	17.60	1478	[8]
$Ni_{51.2}Ti_{48.8}$ (at.%)	15.4	-	-	8.18	722	[9]
$Ni_{50.41}Ti_{49.20}$ (at.%)	35.6	-	-	9.22	976	[9]
$Ni_{50.31}Ti_{49.32}$ (at.%)	65.3	-	-	11.69	1174	[9]
$Ni_{51.2}Ti_{48.8}$ (at.%)	75.1	-	-	10.26	1102	[9]
$Ni_{50.28}Ti_{49.36}$ (at.%)	85.2	-	-	12.52	1352	[9]
$Ni_{50.28}Ti_{49.36}$ (at.%)	78	28.4	6.45	12.92	2366	[10]
$Ni_{51}Ti_{29}Hf_{20}$ (at.%)	52.0	6.6	-	11.19	663	[11]
$Ni_{50.7}Ti_{34.3}Hf_{15}$ (at.%)	76	7.2	-	11.19	653	[11]
$Ni_{51}Ti_{24}Hf_{25}$ (at.%)	124	9.1	-	11.19	955	[11]
$Ni_{51.2}Ti_{18.8}Hf_{30}$ (at.%)	162	14.4	-	11.19	1626	[11]
$Ni_{50.7}Ti_{29.3}Hf_{20}$ (at.%)	170	16.1	-	11.19	1593	[11]
$Ni_{50.3}Ti_{34.7}Hf_{15}$ (at.%)	184	22.6	-	11.19	2098	[11]
$Ni_{50.8}Ti_{29.6}Hf_{19.4}$ (at.%)	193	18.7	-	11.19	1830	[11]
$Ni_{50.3}Ti_{29.7}Hf_{20}$ (at.%)	289	32.5	-	11.19	3217	[11]
$(Ni_{49.5}Mn_{44.5}Ti_{6})_{99.8}B_{0.2}$ (at.%)	492	65.2	7.52	18.47	9056	[39]
$(Ni_{49.5}Mn_{43.5}Ti_{7})_{99.8}B_{0.2}$ (at.%)	438	61.3	7.47	18.09	8284	[39]
$(Ni_{49.5}Mn_{42.5}Ti_{8})_{99.8}B_{0.2}$ (at.%)	412	57.1	7.47	18.00	7678	[39]
$(Ni_{49.5}Mn_{41.5}Ti_{9})_{99.8}B_{0.2}$ (at.%)	372	52.8	7.44	18.00	7071	[39]
$(Ni_{49.5}Mn_{40.5}Ti_{10})_{99.8}B_{0.2}$ (at.%)	330	48.4	7.43	18.00	6473	[39]
$(Ni_{49.5}Mn_{39.5}Ti_{11})_{99.8}B_{0.2}$ (at.%)	292	44.6	7.39	18.66	6150	[39]
$Cu_{83.4}Al_{13.2}Ni_{3.4}$ (wt.%)	142	8.47	6.356	101	5437	[40]
$Ti_{50.2}Ni_{43.6}Cu_{5.8}Al_{0.4}$ (at.%)	/	/	/	/	2690	[41]
$Ni_{51}Ti_{24}Hf_{25}$ (at.%)	124	9.1	9.38	11.19	955	[46]
$Ni_{51.2}Ti_{18.8}Hf_{30}$ (at.%)	162	14.4	10.09	11.19	1626	[46]
$Ni_{50.7}Ti_{29.3}Hf_{20}$ (at.%)	170	16.1	8.842	11.19	1593	[46]
$Ni_{50.3}Ti_{34.7}Hf_{15}$ (at.%)	184	22.6	8.296	11.19	2098	[46]
$Ni_{50.8}Ti_{29.6}Hf_{19.4}$ (at.%)	193	18.7	8.745	11.19	1830	[46]
$Ni_{50.3}Ti_{29.7}Hf_{20}$ (at.%)	289	32.5	8.85	11.19	3217	[46]
$Ni_{50.7}Ti_{24.3}Hf_{25}$ (at.%)	334	16.3	9.52	11.19	1737	[46]
$Ni_{50.3}Ti_{24.7}Hf_{25}$ (at.%)	440	20.1	9.26	11.19	2083	[46]
$Ni_{50.3}Ti_{19.7}Hf_{30}$ (at.%)	622	12.2	10.55	11.19	1440	[46]
$Ti_{30}Ni_{47}Hf_{8}Zr_{15}$ (at.%)	371.13	36.1	7.56	22.38	6104	[47]
$Ti_{31.1}Ni_{47}Hf_{6.9}Zr_{15}$ (at.%)	340.67	35.5	7.4	20.71	5436	[47]
$Ti_{27.2}Ni_{47}Hf_{13.8}Zr_{12}$ (at.%)	461.21	37.1	8.16	15.68	4750	[47]
$Ti_{30.5}Ni_{47}Hf_{7.5}Zr_{15}$ (at.%)	357.31	35.1	7.52	22.74	6003	[47]
$Ti_{29.5}Ni_{47}Hf_{8.5}Zr_{15}$ (at.%)	383.01	36.9	7.6	19.73	5531	[47]
$Ti_{31}Ni_{47}Hf_{12.4}Zr_{9.6}$ (at.%)	333.76	35	7.91	22.43	6208	[47]
$Ti_{25.8}Ni_{47}Hf_{12.4}Zr_{14.8}$ (at.%)	509.59	36.4	8.05	18.71	5485	[47]
$Ti_{25.7}Ni_{47}Hf_{12.4}Zr_{14.9}$ (at.%)	508.51	34.4	8.04	16.45	4549	[47]
$Ti_{29.9}Ni_{47}Hf_{12.3}Zr_{10.8}$ (at.%)	505.49	34.5	8.05	19.48	5410	[47]
$Ti_{30}Zr_{5}Hf_{20}Ni_{35}Cu_{10}$ (at.%)	297	15.9	8.63	16.6	2278	[48]

$Ti_{28}Zr_7Hf_{20}Ni_{35}Cu_{10}$ (at.%)	349	17.1	8.66	16.6	2458	[48]
$Ti_{26}Zr_9Hf_{20}Ni_{35}Cu_{10}$ (at.%)	413	18.7	8.67	16.6	2691	[48]
$Ti_{24}Zr_{11}Hf_{20}Ni_{35}Cu_{10}$ (at.%)	469	20.2	8.69	16.6	2914	[48]
$(Ni_{49.5}Mn_{44.5}Ti_6)_{99.8}B_{0.2}$ (at.%)	492	65.2	7.52	18.47	9056	[49]
$(Ni_{49.5}Mn_{43.5}Ti_7)_{99.8}B_{0.2}$ (at.%)	438	61.3	7.47	18.09	8284	[49]
$(Ni_{49.5}Mn_{42.5}Ti_8)_{99.8}B_{0.2}$ (at.%)	412	57.1	7.47	18	7678	[49]
$(Ni_{49.5}Mn_{41.5}Ti_9)_{99.8}B_{0.2}$ (at.%)	372	52.8	7.44	18	7071	[49]
$(Ni_{49.5}Mn_{40.5}Ti_{10})_{99.8}B_{0.2}$ (at.%)	330	48.4	7.43	18	6473	[49]
$(Ni_{49.5}Mn_{39.5}Ti_{11})_{99.8}B_{0.2}$ (at.%)	292	44.6	7.39	18.66	6150	[49]
$Cu_{83.4}Al_{13.2}Ni_{3.4}$(wt%) (at.%)	142	8.47	6.356	101	5437	[50]
$Ni_{47.73}Ti_{52.27}$ (at.%)	57	13.5	6.239	17.6	1478	[51]
$Ni_{51.2}Ti_{48.8}$ (at.%)	23.9	6.95	NA	8.18	722	[52]
$Ni_{50.41}Ti_{49.20}$ (at.%)	35.6	14.68	NA	9.22	976	[52]
$Ni_{50.31}Ti_{49.32}$ (at.%)	65.3	20.51	NA	11.69	1174	[52]
$Ni_{51.2}Ti_{48.8}$ (at.%)	80.9	19.19	NA	10.26	1102	[52]
$Ni_{50.28}Ti_{49.36}$ (at.%)	85.2	28.56	NA	12.52	1352	[52]
$Ni_{50.28}Ti_{49.36}$ (at.%)	78	28.4	6.45	12.92	2366	[53]
$Ni_{51}Ti_{29}Hf_{20}$ (at.%)	52	6.6	8.98	11.19	663	[46]
$Ni_{50.7}Ti_{34.3}Hf_{15}$ (at.%)	76	7.2	8.1	11.19	653	[46]
$Ti_{50.4}Ni_{43.4}Cu_{5.8}Nb_{0.4}$ (at.%)	67.87	27.52	6.32	10.8	1878.41	[54]
$Ti_{50.6}Ni_{43}Cu_{6.2}Cr_{0.2}$ (at.%)	63.22	27.26	6.32	11.96	2060.51	[54]
$Ti_{50.2}Ni_{43.6}Cu_{5.8}V_{0.4}$ (at.%)	64.63	28.41	6.41	10.69	1946.74	[54]
$Ti_{50.2}Ni_{43.6}Cu_{5.8}Al_{0.4}$ (at.%)	81.37	30.24	6.39	13.92	2689.81	[54]
Cu_{84} Al_{13} Ni_3 (wt.%)	163	9.86	7.199	102	7272	This work
$Cu_{83.9}$ $Al_{13.1}$ Ni_3 (wt.%)	143	9.5	/	/	/	This work
$Cu_{83.8}$ $Al_{13.2}$ Ni_3 (wt.%)	137	9.34	/	/	/	This work
$Cu_{83.7}$ $Al_{13.2}$ $Ni_{3.1}$ (wt.%)	125	9.27	/	/	/	This work
**Organic SL-PCMs (commercial materials)**						
PlusICE A2	2	230	0.765	0.21	37	[1]
PlusICE A3	3	230	0.765	0.21	37	[1]
PlusICE A4	4	235	0.766	0.21	38	[1]
PlusICE A5	5	170	0.768	0.22	29	[1]
PlusICE A6	6	185	0.768	0.21	30	[1]
PlusICE A6.5	6.5	190	0.77	0.22	32	[1]
PlusICE A7	7	190	0.77	0.22	32	[1]
PlusICE A8	8	180	0.77	0.21	29	[1]
PlusICE A9	9	190	0.77	0.21	31	[1]
PlusICE A10	10	210	0.77	0.22	36	[1]
PlusICE A11	11	210	0.775	0.22	36	[1]
PlusICE A12	12	215	0.775	0.22	37	[1]
PlusICE A13	13	225	0.775	0.22	38	[1]
PlusICE A14	14	200	0.775	0.22	34	[1]

PlusICE A15	15	205	0.78	0.18	29	[1]
PlusICE A16	16	225	0.78	0.18	32	[1]
PlusICE A17	17	235	0.78	0.18	33	[1]
PlusICE A18	18	155	0.765	0.22	26	[1]
PlusICE A19	19	150	0.765	0.22	25	[1]
PlusICE A20	20	160	0.77	0.22	27	[1]
PlusICE A21	21	160	0.77	0.22	27	[1]
PlusICE A22	22	160	0.785	0.18	23	[1]
PlusICE A23	23	155	0.785	0.18	22	[1]
PlusICE A24	24	155	0.79	0.18	22	[1]
PlusICE A25	25	220	0.785	0.18	31	[1]
PlusICE A26	26	230	0.79	0.21	38	[1]
PlusICE A27	27	250	0.768	0.22	42	[1]
PlusICE A28	28	265	0.789	0.21	44	[1]
PlusICE A29	29	230	0.785	0.21	38	[1]
PlusICE A30	30	230	0.79	0.21	38	[1]
PlusICE A31	31	230	0.79	0.21	38	[1]
PlusICE A32	32	120	0.845	0.21	21	[1]
PlusICE A33	33	230	0.79	0.21	38	[1]
PlusICE A34	34	230	0.79	0.21	38	[1]
PlusICE A36	36	250	0.776	0.22	43	[1]
PlusICE A39	39	135	0.9	0.22	27	[1]
PlusICE A42	42	140	0.905	0.21	27	[1]
PlusICE A43	43	280	0.78	0.18	39	[1]
PlusICE A46	46	155	0.91	0.22	31	[1]
PlusICE A48	48	230	0.81	0.18	36	[1]
PlusICE A53	53	155	0.91	0.22	31	[1]
PlusICE A58	58	240	0.82	0.18	35	[1]
PlusICE A62	62	205	0.91	0.22	41	[1]
PlusICE A70	70	225	0.89	0.23	46	[1]
PlusICE A73	73	225	0.89	0.23	46	[1]
PlusICE A78	78	225	0.89	0.23	46	[1]
PlusICE A82	82	240	0.93	0.23	51	[1]
PlusICE A95	95	260	0.9	0.22	51	[1]
PlusICE A118	118	195	0.9	0.22	39	[1]
PlusICE A133	133	200	0.88	0.23	40	[1]
PlusICE A144	144	115	0.88	0.23	23	[1]
PlusICE A155	155	102	0.88	0.23	21	[1]
RT 2 HC	1-3	200	0.77	0.2	30.8	[12]
RT 3 HC_1	1-3	190	0.77	0.2	29.26	[12]
RT 4	2-4	175	0.77	0.2	26.95	[12]
RT 5	5-7	180	0.77	0.2	27.72	[12]

RT 5 HC	5-6	250	0.76	0.2	38	[12]
RT 8	6-9	175	0.77	0.2	26.95	[12]
RT 8 HC	7-9	190	0.77	0.2	29.26	[12]
RT 10	4-10	160	0.77	0.2	24.64	[12]
RT 10 HC	9-10	200	0.77	0.2	30.8	[12]
RT 11 HC	10-12	200	0.77	0.2	30.8	[12]
RT 12	7-13	155	0.77	0.2	23.87	[12]
RT 15	10-17	155	0.77	0.2	23.87	[12]
RT 18 HC	17-19	260	0.77	0.2	40.04	[12]
RT 21	18-23	155	0.77	0.2	23.87	[12]
RT 21HC	20-23	190	0.77	0.2	29.26	[12]
RT 22HC	20-23	190	0.7	0.2	26.6	[12]
RT 24	21-25	160	0.77	0.2	24.64	[12]
RT 25 HC	22-26	210	0.77	0.2	32.34	[12]
RT 26	25-26	180	0.75	0.2	27	[12]
RT 28HC	27-29	250	0.77	0.2	38.5	[12]
RT 31	27-33	165	0.76	0.2	25.08	[12]
RT 35	29-36	160	0.77	0.2	24.64	[12]
RT 35 HC	34-36	240	0.77	0.2	36.96	[12]
RT 42	38-43	165	0.76	0.2	25.08	[12]
RT 44HC	41-44	250	0.7	0.2	35	[12]
RT 47	41-48	160	0.77	0.2	24.64	[12]
RT 50	45-51	160	0.76	0.2	24.32	[12]
RT 54HC	53-54	200	0.8	0.2	32	[12]
RT 55	51-57	170	0.77	0.2	26.18	[12]
RT 60	55-61	160	0.77	0.2	24.64	[12]
RT 62HC	62-63	230	0.84	0.2	38.64	[12]
RT 64HC	63-65	250	0.78	0.2	39	[12]
RT 65	58-65	150	0.78	0.2	23.4	[12]
RT 69HC	68-70	230	0.84	0.2	38.64	[12]
RT 70HC	69-71	260	0.77	0.2	40.04	[12]
RT 82	77-82	170	0.77	0.2	26.18	[12]
RT 80 HC	77-80	220	0.8	0.2	35.2	[12]
RT 90 HC	91-92	170	0.85	0.2	28.9	[12]
RT 100	90-112	120	0.77	0.2	18.48	[12]
RT 100 HC	99-101	180	0.85	0.2	30.6	[12]
RT 111 HC	110-112	210	1	0.2	42	[12]
**Organic SL-PCMs (non-commercial materials)**						
Paraffin	40-43	189	0.765	0.29	42	[13]
Paraffin wax	64	173.6	0.79	0.167	23	[14]
Medicinal paraffin	44	146	0.83	2.1	254	[15]
Commercial paraffin wax	52	244	0.8095	0.15	30	[15]

Paraffin 56	56	86	1.06	0.75	68	[16]
Paraffin natural wax 79	79	80	1.2	0.63	60	[16]
Paraffin natural wax 84	84	85	1.2	0.72	73	[16]
Paraffin natural wax 106	106	80	1.2	0.65	62	[16]
Paraffin 53	53	164	0.795	0.19	25	[16]
RT 60 Rubitherm paraffin	60	214	0.775	0.2	33	[16]
Paraffin C_{13}-C_{24}	24	189	0.00076	0.21	0.03	[18, 19]
Paraffin C_{18}	28	244	0.000774	0.15	0.03	[18, 19]
Paraffin C_{16}-C_{28}	44	189	0.000765	0.21	0.03	[18, 19]
Paraffin C_{20}-C_{33}	50	189	0.000769	0.21	0.03	[18, 19]
Paraffin C_{22}-C_{45}	60	189	0.000795	0.21	0.03	[18, 19]
Paraffin C_{23}-C_{45}	64	189	0.000915	0.21	0.04	[18]
Paraffin C_{21}-C_{50}	68	189	0.00083	0.21	0.03	[18, 19]
Capric acid	32	152.7	0.878	0.153	21	[14]
Myristic acid	51.5	204.5	0.861	0.15	26	[19]
Palmitic acid	64	185.4	0.85	0.162	26	[17]
Caprylic acid	16.3	148	0.901	0.149	20	[20]
Lauric acid	42.6	211.6	0.862	0.147	27	[19]
Stearic acid	71	210	0.848	0.172	31	[2]
Formic acid	8	277	1.227	0.27	92	[21]
Acetic acid	17	192	1.214	0.19	44	[21]
Benzoic acid	121.7	142.8	1.266	0.139	25	[22]
1-Octadecanol	60	225	0.81	0.15	27	[10]
N-eicosane	36.8	247	0.788	0.15	29	[13]
n-Heptadecane	19	240	0.76	0.21	38	[23]
n-Octadecane	27.7	243.5	0.771	0.148	28	[24]
n-Dodecane	-12	216	0.75	0.21	34	[20]
n-Pentadecane	10	207	0.768	0.17	27	[20]
n-Hexadecane	18.2	238	0.774	0.21	39	[20]
n-Nonadecane	31.9	222	0.769	0.21	36	[20]
n-Hexacosane	56	257	0.77	0.21	42	[20]
Dimethyl adipate	9.7	164.6	1.062	0.358	63	[20]
Tetradecane	5.8	228	0.825	0.15	28	[2]
Erythritol	118	339.8	1.3	0.326	144	[17]
Napthalene	80	147.7	0.976	0.132	19	[2]
Acetamide	82	260	1.16	0.25	75	[21]
HDPE	130	255	0.952	0.44	107	[21]
Urea	134	250	1.32	0.6	198	[21]
d-Mannitol	165	300	1.49	0.11	49	[21]
Xylitol	94	263	1.5	0.412	163	[22]
Catechol	104.3	207	1.37	0.04	11	[22]
Acetanilide	115	142	1.21	0.35	60	[22]

Polyglycol 400	8	99.6	1.125	0.187	21	[2]
Polyglycol E600	22	127.2	1.126	0.189	27	[17]
Polyglycol 900	34	150.5	1.1	0.188	31	[2]
Polyethylene glycol	20	146	1.1	0.19	31	[25]
**Hydrated salt SL-PCMs (commercial materials)**						
PlusICE S8	8	130	1.475	0.44	84	[1]
PlusICE S10	10	170	1.47	0.43	107	[1]
PlusICE S13	13	150	1.515	0.43	98	[1]
PlusICE S15	15	180	1.51	0.43	117	[1]
PlusICE S17	17	155	1.525	0.43	102	[1]
PlusICE S18	18	145	1.52	0.43	95	[1]
PlusICE S19	19	175	1.52	0.43	114	[1]
PlusICE S20	20	195	1.53	0.54	161	[1]
PlusICE S21	21	220	1.53	0.54	182	[1]
PlusICE S22	22	215	1.53	0.54	178	[1]
PlusICE S23	23	200	1.53	0.54	165	[1]
PlusICE S24	24	180	1.53	0.54	149	[1]
PlusICE S25	25	175	1.53	0.54	145	[1]
PlusICE S27	27	185	1.53	0.54	153	[1]
PlusICE S32	32	220	1.46	0.51	164	[1]
PlusICE S34	34	140	2.1	0.52	153	[1]
PlusICE S46	46	110	1.61	0.6	106	[1]
PlusICE S47	47	110	1.61	0.6	106	[1]
PlusICE S48	48	110	1.565	0.61	105	[1]
PlusICE S49	49	110	1.56	0.61	105	[1]
PlusICE S50	50	110	1.545	0.62	105	[1]
PlusICE S51	51	110	1.515	0.63	105	[1]
PlusICE S52	52	115	1.512	0.63	110	[1]
PlusICE S53	53	115	1.51	0.65	113	[1]
PlusICE S54	54	120	1.51	0.65	118	[1]
PlusICE S55	55	120	1.508	0.65	118	[1]
PlusICE S56	56	130	1.505	0.67	131	[1]
PlusICE S57	57	135	1.505	0.67	136	[1]
PlusICE S58	58	145	1.505	0.69	151	[1]
PlusICE S70	70	100	1.68	0.57	96	[1]
PlusICE S72	72	155	1.666	0.58	150	[1]
PlusICE S83	83	100	1.6	0.62	99	[1]
PlusICE S89	89	145	1.55	0.67	151	[1]
PlusICE S117	117	125	1.45	0.7	127	[1]
SP 5_gel	2-7	155	1.3	0.6	121	[12]
SP 7_gel	5-8	155	1.3	0.6	121	[12]
SP 9_gel	10-11	155	1.3	0.6	121	[12]

SP 11_gel	12-13	155	1.32	0.6	123	[12]
SP 15_gel	15-17	160	1.35	0.6	130	[12]
SP 21 EK	21-23	170	1.4	0.6	143	[12]
SP 24 E	24-25	180	1.4	0.5	126	[12]
SP 25 E2	24-26	180	1.4	0.5	126	[12]
SP 26E	25-27	180	1.4	0.5	126	[12]
SP 29 Eu	29-31	200	1.5	0.5	150	[12]
SP 31	31-33	210	1.3	0.5	137	[12]
SP 50	50-51	220	1.3	0.6	172	[12]
SP 58	56-59	250	1.3	0.6	195	[12]
SP 70	67-73	150	1.3	0.6	117	[12]
SP 90	88-90	150	1.65	0.6	149	[12]
**Hydrated salt SL-PCMs (non-commercial materials)**						
$MgCl_2{\cdot}6H_2O$ (at.%)	117	168.7	1.45	0.57	139	[14]
$Mg(NO_3)_2{\cdot}6H_2O$ (at.%)	89	162.8	1.55	0.49	124	[15, 27]
$Ba(OH)_2{\cdot}8H_2O$ (at.%)	78	265.7	1.937	0.653	336	[15, 27]
$Na_2SO_4{\cdot}10H_2O$ (at.%)	32.4	254	1.485	0.544	205	[17]
$LiNO_3{\cdot}3H_2O$ (at.%)	30.1	284	1.42	0.58	234	[27]
$CaCl_2{\cdot}12H_2O$ (at.%)	29.8	174	1.53	0.53	141	[20]
$KF{\cdot}4H_2O$ (at.%)	18.4	246	1.45	0.48	171	[28]
$CaCl_2{\cdot}6H_2O$ (at.%)	30	200	1.8	0.54	194	[29]
$Na_2HPO_4{\cdot}12H_2O$ (at.%)	45	279.6	1.52	0.476	202	[29]
$MgCl_2{\cdot}6H_2O$ (at.%)	117	168.6	1.45	0.57	139	[23]
$Zn(NO_3)_2{\cdot}6H_2O$ (at.%)	36	146.9	1.828	0.464	125	[30]
$Na_2S_2O_3{\cdot}5H_2O$ (at.%)	46	210	1.666	0.38	133	[31]
$Na_2SO_4{\cdot}10H_2O$ (at.%)	32	180	1.485	0.45	120	[31]
$C_2H_2O_4{\cdot}2H_2O$ (at.%)	105	264	1.653	0.7	305	[31]
$CH_3COONa{\cdot}3H_2O$ (at.%)	58	264	1.28	0.63	213	[23]
Inorganic salt SL-PCMs (non-commercial materials)						
$NaNO_3$ (at.%)	306	172	2.261	0.5	194	[32]
KNO_3 (at.%)	335	95	2.109	0.5	100	[33, 34]
KOH (at.%)	360	134	2.04	0.5	137	[32]
Na_2CO_3 (at.%)	854	276	2.533	2	1398	[34]
K_2CO_3 (at.%)	897	236	2.29	2	1081	[34]
$ZnCl_2$ (at.%)	280	75	2.907	0.5	109	[15]
NaOH (at.%)	318	165	2.1	0.92	319	[15]
LiH (at.%)	685	2580	0.55	2.1	2980	[35]
Li_2CO_3 (at.%)	726	607	2.11	1.96	2510	[35]
$MgCl_2$ (at.%)	714	452	2.14	1.17	1132	[35]
**Traditional high-temperature SL-PCMs (commercial materials)**						
PlusICE H105	104	125	1.7	0.5	107	[1]
PlusICE H115	114	100	2.2	0.5	110	[1]

PlusICE H120	120	120	2.22	0.51	136	[1]
PlusICE H160	162	105	1.91	0.51	103	[1]
PlusICE H190	191	170	2.3	0.51	199	[1]
PlusICE H220	220	100	2	0.52	104	[1]
PlusICE H230	227	105	1.55	0.52	85	[1]
PlusICE H250	250	280	2.38	0.52	346	[1]
PlusICE H255	254	270	2.38	0.52	334	[1]
PlusICE H280	282	160	2.25	0.53	191	[1]
PlusICE H285	285	85	2.2	0.53	99	[1]
PlusICE H290	292	150	2.2	0.53	175	[1]
PlusICE H300	302	130	1.9	0.54	133	[1]
PlusICE H305	305	150	1.57	0.54	127	[1]
PlusICE H320	320	70	2.1	0.55	81	[1]
PlusICE H325	327	80	2.11	0.55	93	[1]
PlusICE H335	334	80	2.11	0.55	93	[1]
PlusICE H355	353	230	2.06	0.56	265	[1]
PlusICE H380	382	225	2.05	0.56	258	[1]
PlusICE H395	395	215	2.33	0.56	281	[1]
PlusICE H425	425	220	2.1	0.57	263	[1]
PlusICE H430	430	125	2.16	0.57	154	[1]
PlusICE H485	483	200	2.22	0.57	253	[1]
PlusICE H500	500	300	2.22	0.57	380	[1]
PlusICE H500A	500	140	2.14	0.57	171	[1]
PlusICE H525	525	155	2.35	0.57	207	[1]
PlusICE H535	535	130	2.32	0.56	169	[1]
PlusICE H610	610	410	2.07	0.56	475	[1]
PlusICE H640	640	338	2.38	0.56	450	[1]
PlusICE H650	652	300	2.45	0.56	412	[1]
PlusICE H690	687	250	2.4	0.56	336	[1]
PlusICE H695	695	280	2.46	0.56	386	[1]
PlusICE H700	699	250	2.41	0.57	344	[1]
PlusICE H705	706	250	2.43	0.57	347	[1]
PlusICE H705A	705	452	2.04	0.57	526	[1]
PlusICE H755	755	466	2.16	0.58	584	[1]
PlusICE H845	845	276	2.53	0.59	412	[1]
PlusICE H885	885	236	2.29	0.59	319	[1]
**Organic–organic composite SL-PCMs (non-commercial materials)**						
38.5trimethyloletane-31.5water-30urea (wt.%)	14.4	160	1.17	0.66	124	[23]
34Mistiric acid-66Capric acid (wt.%)	24	147.7	0.888	0.164	22	[17]
38Urea-62acetamide (wt.%)	53	224	1.216	0.34	93	[21]
83Stearic acid-17acetamide (wt.%)	65	213	0.972	0.18	37	[21]

36Stearic acid-64palmitic acid (wt.%)	53	182	0.971	0.169	30	[31]
**Inorganic–inorganic composite SL-PCMs (non-commercial materials)**						
61.5Mg$(NO_3)_2$·6H_2O-38.5NH_4NO_3 (wt.%)	52	125.5	1.515	0.515	98	[2]
58.7Mg$(NO_3)_2$·6H_2O-41.3$MgCl_2$·6H_2O (wt.%)	59	132.2	1.55	0.565	116	[2]
50Mg$(NO_3)_2$·6H_2O-50$MgCl_2$·6H_2O (wt.%)	58	132	1.55	0.51	104	[23]
58.7Mg$(NO_3)_2$·6H_2O-41.3$MgCl_2$·6H_2O (wt.%)	59	132.2	1.55	0.51	105	[23]
67$CaCl_2$·6H_2O-33$MgCl_2$·6H_2O (wt.%)	25	127	1.661	0.55	116	[21]
59Mg$(NO_3)_2$·6H_2O-41$MgCl_2$·6H_2O (wt.%)	59	132	1.61	0.53	113	[21]
14$LiNO_3$-86$MgNO_3$·6H_2O (wt.%)	72	180	1.713	0.51	157	[21]
50KCl-50$LiNO_3$ (wt.%)	165.6	201.7	2.01	1.749	709	[36]
60$NaNO_3$-40KNO_3 (wt.%)	223.2	142.2	1.899	2.272	614	[36]
95.5KNO_3-4.5KCl (wt.%)	320	74	2.1	0.5	78	[36]
54KCl-46$ZnCl_2$ (wt.%)	432	218	2.41	0.83	436	[37]
61KCl-39$MgCl_2$ (wt.%)	435	351	2.11	0.81	600	[37]
48NaCl-52$MgCl_2$ (wt.%)	450	430	2.23	0.95	911	[37]
36KC_1-64$MgCl_2$ (wt.%)	470	388	2.19	0.83	705	[37]
33NaCl-67$CaCl_2$ (wt.%)	500	281	2.16	1.02	619	[37]
37$MgCl_2$-63$SrCl_2$ (wt.%)	535	239	2.78	1.05	698	[37]
47Li_2CO_3-53K_2CO_3 (wt.%)	488	342	2.2	1.99	1497	[37]
44Li_2CO_3-56Na_2CO_3 (wt.%)	496	370	2.32	2.09	1794	[37]
28Li_2CO_3-72K_2CO_3 (wt.%)	498	263	2.24	1.85	1090	[37]
51K_2CO_3-49Na_2CO_3 (wt.%)	710	163	2.4	1.73	677	[37]
45NaBr-55$MgBr_2$ (wt.%)	431	212	3.49	0.9	666	[37]
25KC_1-27$CaCl_2$-48$MgCl_2$ (wt.%)	487	342	2.53	0.88	761	[37]
5KCl-29NaCl-66$CaCl_2$ (wt.%)	504	279	2.15	1	600	[37]
13KCl-19NaCl-68$SrCl_2$ (wt.%)	504	223	2.75	1.05	644	[37]
28KC_1-19NaCl-53$BaCl_2$ (wt.%)	542	221	3.02	0.86	574	[37]
24KCl-47$BaCl_2$-29$CaCl_2$ (wt.%)	551	219	2.93	0.95	610	[37]
32Li_2CO_3-35K_2CO_3-33Na_2CO_3 (wt.%)	397	276	2.3	2.02	1282	[37]
40KC_1-23KF-37K_2CO_3 (wt.%)	528	283	2.28	1.19	768	[37]
17NaF-21KF-62K_2CO_3 (wt.%)	520	274	2.38	1.5	978	[37]
35Li_2CO_3-65K_2CO_3 (wt.%)	505	344	2.26	1.89	1469	[37]
20Li_2CO_3-60Na_2CO_3-20K_2CO_3 (wt.%)	550	283	2.38	1.83	1233	[37]
22Li_2CO_3-16Na_2CO_3-62K_2CO_3 (wt.%)	580	288	2.34	1.95	1314	[37]
46LiF-44NaF_2-10MgF_2 (wt.%)	632	858	2.24	1.2	2306	[37]
50NaCl-50$MgCl_2$ (wt.%)	450	429	2.24	0.96	923	[37]
31Li_2CO_3-35K_2CO_3-34Na_2CO_3 (wt.%)	397	275	2.31	2.04	1296	[37]
63$MgCl_2$-23NaCl-14KCl (wt.%)	385	461	2.25	0.95	985	[37]
37LiCl-63LiOH (wt.%)	262	485	1.55	1.1	827	[37]
50$NaNO_3$-50KNO_3 (wt.%)	220	100.7	1.92	0.56	108	[15]

68.1KCl-31.9ZnCl$_2$ (wt.%)	235	198	2.48	0.8	393	[2]
61.5Mg(NO$_3$)$_2$-38.5NH$_4$NO$_3$ (wt.%)	52	125.5	1.515	0.495	94	[23]
56KNO$_3$-44NaNO$_2$ (wt.%)	141	97	1.994	0.57	110	[21]
53KNO$_3$-6NaNO$_3$-41NaNO$_2$ (wt.%)	142	110	2.006	0.57	126	[21]
48KNO$_2$-52NaNO$_3$ (wt.%)	149	124	2.08	0.52	134	[21]
62LiNO$_3$-38NaNO$_2$ (wt.%)	156	233	2.296	0.66	353	[21]
58LiNO$_3$-42KCl (wt.%)	160	272	2.196	0.59	352	[21]
45LiNO$_3$-50NaNO$_3$-5KCl (wt.%)	160	266	2.297	0.59	360	[21]
19LiOH-81LiNO$_3$ (wt.%)	183	352	2.124	0.69	516	[21]
49LiNO$_3$-51NaNO$_3$ (wt.%)	194	262	2.317	0.59	358	[21]
87LiNO$_3$-13NaCl (wt.%)	208	369	2.35	0.63	546	[21]
80KNO$_3$-20KOH (wt.%)	214	83	1.905	0.54	85	[21]
55KNO$_3$-45NaNO$_3$ (wt.%)	222	110	2.028	0.51	114	[21]
27LiBr-73LiNO$_3$ (wt.%)	228	279	2.603	0.57	414	[21]
6LiOH-67NaNO$_3$-27NaOH (wt.%)	230	184	2.154	0.67	266	[21]
55NaNO$_2$-45NaNO$_3$ (wt.%)	233	163	2.21	0.64	231	[21]
13CaCl$_2$-87LiNO$_3$ (wt.%)	238	317	2.362	0.69	517	[21]
9LiCl-91LiNO$_3$ (wt.%)	244	342	2.351	0.64	515	[21]
86NaNO$_3$-14NaOH (wt.%)	250	160	2.241	0.6	215	[21]
58NaCl-42KCl (wt.%)	360	119	2.0844	0.48	119	[22]
41.69Na$_2$CO$_3$-33.1KCl-25.21NaCl (wt.%)	569	249.6	1.7	0.5	212	[38]
40.55NaCl-59.45Na$_2$CO$_3$ (wt.%)	635	311	2.07	0.5	322	[38]
80.5LiF-19.5CaF$_2$ (wt.%)	767	816	2.39	1.7	3315	[38]
60.2LiF-39.8CaF$_2$ (wt.%)	767	815	2.1	1.6	2738	[35]
57.8LiF-42.2CaF$_2$ (wt.%)	769	816	2.39	1.7	3315	[35]
40KCl-37K$_2$CO$_3$-23KF (wt.%)	528	283	2.28	1.19	768	[35]
54.3KNO$_3$-45.7NaNO$_3$ (wt.%)	220	101	1.92	0.56	109	[35]
55.8NaNO$_3$-44.2KNO$_3$ (wt.%)	220	109	1.8	0.8	156.96	[35]
34.5K$_2$CO$_3$-33.4Na$_2$CO$_3$- 32.1Li$_2$CO$_3$ (wt.%)	397	276	2.3	2.02	1282	[35]
55.6Na$_2$CO$_3$-44.4Li$_2$CO$_3$ (wt.%)	496	370	2.32	2.09	1794	[35]
72Na$_2$CO$_3$-28Li$_2$CO$_3$ (wt.%)	498	263	2.24	1.85	1090	[35]
50K$_2$CO$_3$-50Na$_2$CO$_3$ (wt.%)	710	163	2.4	1.73	677	[35]
**Organic–inorganic composite SL-PCMs (non-commercial materials)**						
60Urea-40CH$_3$COONa·3H$_2$O (wt.%)	30	200	1.37	0.48	132	[21]
61Mg(NO$_3$)$_2$·6H$_2$O-39NH$_4$NO$_3$(wt.%)	52	125	1.672	0.5	105	[21]
82Urea-18LiNO$_3$ (wt.%)	76	218	1.438	0.6	188	[21]
71Urea-29NaNO$_3$ (wt.%)	83	187	1.502	0.59	166	[21]
85Urea-15NH$_4$Cl (wt.%)	102	214	1.348	0.58	167	[21]
15Urea-85K$_2$CO$_3$ (wt.%)	102	206	1.415	0.58	169	[21]
77Urea-23KNO$_3$ (wt.%)	109	195	1.416	0.58	160	[21]
90Urea-10NaCl (wt.%)	112	230	1.372	0.6	189	[21]

89Urea-11KCl (wt.%)	115	227	1.37	0.66	205	[21]

Software:

All machine learning models were implemented using Python 3.9.18 and R 4.4.0. The RFR, XGBR, and LGBR models were implemented using the scikit-learn, XGBoost, and LightGBM libraries, respectively, while the noise-aware Kriging model was implemented using the DiceKriging package in R.

Reference

[1] Phase change material products limited-PlusICE phase change materials. Available from: https://www.pcmproducts.net/files/PlusICE%20Range%202021-1.pdf.

[2] Jankowski NR, McCluskey FP, A review of phase change materials for vehicle component thermal buffering, Appl. Energy 113 (2014) 1525-1561.

[3] Kenisarin MM, Thermophysical properties of some organic phase change materials for latent heat storage. A review, Sol. Energy 107 (2014) 553-575.

[4] Li W, Zhang D, Zhang T, Wang T, Ruan D, Xing D, Li H, Study of solid–solid phase change of (n-CnH2n+1NH3)2MCl4 for thermal energy storage, Thermochim. Acta 326 (1999) 183-186.

[5] Busico V, Corradini P, Vacatello M, Fittipaldi F, Nicolais L. Solid-solid phase transitions for thermal energy storage, 1981. C. den Ouden, Thermal Storage of Solar Energy: Proceedings of an International TNO-Symposium Held in Amsterdam, The Netherlands, 5–6 November, 1980.

[6] He D, Di Y, Tan Z, Yi F, Dan W, Liu Y, Crystal structures and thermochemistry on phase change materials (n-CnH2n+1NH3)2CuCl4(s) (n=14 and 15), Sol. Energy Mater. Sol. Cells 95 (2011) 2897-2906.

[7] Sari A, Alkan C, Lafci O, Synthesis and thermal properties of poly(styrene-co-ally alcohol)-graft-stearic acid copolymers as novel solid-solid PCMs for thermal energy storage, Sol. Energy 86 (2012) 2282-2292.

[8] Sharar DJ, Donovan BF, Warzoha RJ, Wilson AA, Leff AC, Hanrahan BM, Solid-state thermal energy storage using reversible martensitic transformations, Appl. Phys. Lett. 114 (2019) 143902.

[9] Sharar DJ, Wilson AA, Leff A, Smith A, Atli KC, Elwany A, Arroyave R, Karaman I, Additively manufacturing nitinol as a solid-state phase change material, 2020 19th IEEE Intersociety Conference on Thermal and Thermomechanical Phenomena in Electronic Systems (ITherm) (2020) DOI: 10.1109/ITherm45881.42020.9190336.

[10] Sharar DJ, Leff AC, Wilson AA, Smith A, High-capacity high-power thermal energy storage 18 using solid-solid martensitic transformations, Appl. Therm. Eng. 187 (2021) 116490.

[11] Hite N, Sharar D, Trehern W, Umale T, Atli K, Wilson A, Leff A, Karaman I, NiTiHf shape

memory alloys as phase change thermal storage materials, Acta Mater. 218 (2021) 117175.

[12] Rubitherm phase change material products limited-PCM RT and SP-line phase change materials. Available from: https://www.rubitherm.eu/en/productCategories.html.

[13] Ge H, Li H, Mei S, Liu J, Low melting point liquid metal as a new class of phase change material: An emerging frontier in energy area, Renewable Sustainable Energy Rev. 21 (2013) 331-346.

[14] Shao L, Raghavan A, Kim GH, Emurian L, Rosen J, Papaefthymiou MC, Wenisch TF, Martin MMK, Pipe KP, Figure-of-merit for phase-change materials used in thermal management, Int. J. Heat Mass Transfer 101 (2016) 764-771.

[15] Agyenim F, Hewitt N, Eames P, Smyth M, A review of materials, heat transfer and phase change problem formulation for latent heat thermal energy storage systems (LHTESS), Renewable Sustainable Energy Rev. 14 (2010) 615-628.

[16] Kenisarin M, Mahkamov K, Solar energy storage using phase change materials, Renewable Sustainable Energy Rev. 11 (2007) 1913-1965.

[17] Zalba B, Marin JM, Cabeza LF, Mehling H, Review on thermal energy storage with phase change: materials, heat transfer analysis and applications, Appl. Therm. Eng. 23 (2003) 251-283.

[18] Magendran SS, Khan FSA, Mubarak NM, Vaka M, Walvekar R, Khalid M, Abdullah EC, Nizamuddin S, Karri RR, Synthesis of organic phase change materials (PCM) for energy storage applications: A review, Nano-Struct. Nano-Objects 20 (2019) 100399.

[19] Yuan YP, Zhang N, Tao WQ, Cao XL, He YL, Fatty acids as phase change materials: A review, Renewable Sustainable Energy Rev. 29 (2014) 482-498.

[20] Su WG, Darkwa J, Kokogiannakis G, Review of solid-liquid phase change materials and their encapsulation technologies, Renewable Sustainable Energy Rev. 48 (2015) 373-391.

[21] Sarbu I, Dorca A, Review on heat transfer analysis in thermal energy storage using latent heat storage systems and phase change materials, Int. J. Energy Res. 43 (2019) 29-64.

[22] Sinaga R, Darkwa J, Omer SA, Worall M, The microencapsulation, thermal enhancement, and applications of medium and high-melting temperature phase change materials: A review, Int. J. 19 Energy Res. 46 (2022) 10259.

[23] Cabeza LF, Castell A, Barreneche C, de Gracia A, Fernandez AI, Materials used as PCM in thermal energy storage in buildings: A review, Renewable Sustainable Energy Rev. 15 (2011) 1675-1695.

[24] Adine HA, El Qarnia H, Numerical analysis of the thermal behaviour of a shell-and-tube heat storage unit using phase change materials, Appl. Math. Modell. 33 (2009) 2132-2144.

[25] Reddy KS, Mudgal V, Mallick TK, Review of latent heat thermal energy storage for

improved material stability and effective load management, J. Energy Storage 15 (2018) 205-227.

[26] Dixit P, Reddy VJ, Parvate S, Balwani A, Singh J, Maiti TK, Dasari A, Chattopadhyay S, Salt hydrate phase change materials: Current state of art and the road ahead, J. Energy Storage 51 (2022) 104360.

[27] Shamberger PJ, Reid T, Thermophysical properties of Lithium Nitrate Trihydrate from (253 to 353) K, J. Chem. Eng. Data 57 (2012) 1404-1411.

[28] Shamberger PJ, Reid T, Thermophysical properties of potassium fluoride tetrahydrate from (243 to 348) K, J. Chem. Eng. Data 58 (2013) 294-300.

[29] Pielichowska K, Pielichowski K, Phase change materials for thermal energy storage, Prog. Mater. Sci. 65 (2014) 67-123.

[30] Purohit BK, Sistla VS, Inorganic salt hydrate for thermal energy storage application: A review, Energy Storage 3 (2021) e212.

[31] da Cunha JP, Eames P, Thermal energy storage for low and medium temperature applications using phase change materials - A review, Appl. Energy 177 (2016) 227-238.

[32] Michels H, Pitz-Paal R, Cascaded latent heat storage for parabolic trough solar power plants, Sol. Energy 81 (2007) 829-837.

[33] Crespo A, Barreneche C, Ibarra M, Platzer W, Latent thermal energy storage for solar process heat applications at medium-high temperatures - A review, Sol. Energy 192 (2019) 3-34.

[34] Zhang HL, Baeyens J, Caceres G, Degreve J, Lv YQ, Thermal energy storage: Recent developments and practical aspects, Prog. Energy Combust. Sci. 53 (2016) 1-40.

[35] Zhou C, Wu SK, Medium- and high-temperature latent heat thermal energy storage: Material database, system review, and corrosivity assessment, Int. J. Energy Res. 43 (2019) 621-661.

[36] Mohamed SA, Al-Sulaiman FA, Ibrahim NI, Zahir MH, Al-Ahmed A, Saidur R, Yilbas BS, 20 Sahin AZ, A review on current status and challenges of inorganic phase change materials for thermal energy storage systems, Renewable Sustainable Energy Rev. 70 (2017) 1072-1089.

[37] Kenisarin MM, High-temperature phase change materials for thermal energy storage, Renewable Sustainable Energy Rev. 14 (2010) 955-970.

[38] Opolot M, Zhao CR, Liu M, Mancin S, Bruno F, Hooman K, A review of high temperature (≥ 500 °C) latent heat thermal energy storage, Renewable Sustainable Energy Rev. 160 (2022) 112293.

[39] Li S, He L, Lu H, Hao J, Wang D, Shen F, Song C, Liu G, Du P, Wang Y, Cong D, Ultrahigh-performance solid-solid phase change material for efficient, high-temperature

thermal energy storage, Acta Materialia 249 (2023) 118852. https://doi.org/10.1016/j.actamat.2023.118852.

[40] Tan C, Zhang M, Yang J, Wang X, Zhao W, Li J, Zhao Q, Tian X, Ultra-high performance Cu-Al-Ni as phase change material for thermal management of high-power electronic devices, Journal of Energy Storage 113 (2025) 115635.

[41] Tian Y, Hu B, Dang P, Dong S, Sun S, Xue D, Lookman T, Zhang T, Design of shape memory alloys with enhanced thermal management properties via adaptively constrained multi-objective optimization, Acta Materialia (2025), https://doi.org/10.1016/j.actamat.2025.121874.

[42] O. Roustant, D. Ginsbourger, and Y. Deville, Dicekriging, diceoptim: Two r packages for the analysis of computer experiments by kriging-based metamodeling and optimization, Journal of Statistical Software 51, 1–55 (2012).

[43] C. K. Williams and C. E. Rasmussen, Gaussian processes for machine learning, Vol. 2 (MIT press Cambridge, MA, 2006).

[44] W. Trehern, N. Hite, R. Ortiz-Ayala, K. Atli, D. Sharar, A. Wilson, R. Seede, A. Leff, and I. Karaman, Niticu shape memory alloys with ultra-low phase transformation range as solid-state phase change materials, Acta Materialia 260, 119310 (2023).

[45] J. Frenzel, A. Wieczorek, I. Opahle, B. Maaß, R. Drautz, and G. Eggeler, On the effect of alloy composition on martensite start temperatures and latent heats in ni–ti-based shape memory alloys, Acta Materialia 90, 213 (2015).

[46] N. Hite, D. Sharar, W. Trehern, T. Umale, K. Atli, A. Wilson, A. Leff, and I. Karaman, Nitihf shape memory alloys as phase change thermal storage materials, Acta Materialia 218, 117175 (2021).

[47] Y. Xian, P. Dang, Y. Tian, X. Jiang, Y. Zhou, X. Ding, J. Sun, T. Lookman, and D. Xue, Compositional design of multicomponent alloys using reinforcement learning, Acta Materialia 274, 120017 (2024).

[48] G. Liu, S. Li, C. Song, Z. Liu, J. Du, X. Xie, Y. Wang, and D. Cong, High-entropy ti-zr-hf-ni-cu alloys as solid-solid phase change materials for high-temperature thermal energy storage, Intermetallics 166, 108177 (2024).

[49] S. Li, L. He, H. Lu, J. Hao, D. Wang, F. Shen, C. Song, G. Liu, P. Du, Y. Wang, and D. Cong, Ultrahigh-performance solid-solid phase change material for efficient, high-temperature thermal energy storage, Acta Materialia 249, 118852 (2023).

[50] C. Tan, M. Zhang, J. Yang, X. Wang, W. Zhao, J. Li, Q. Zhao, and X. Tian, Ultra-high performance cu-al-ni as phase change material for thermal management of high-power electronic devices, Journal of Energy Storage 113, 115635 (2025).

[51] D. J. Sharar, B. F. Donovan, R. J. Warzoha, A. A. Wilson, A. C. Leff, and B. M. Hanrahan, Solid-state thermal energy storage using reversible martensitic transformations, Applied

Physics Letters 114, 10.1063/1.5087135 (2019).

[52] D. J. Sharar, A. A. Wilson, A. Leff, A. Smith, K. C. Atli, A. Elwany, R. Arroyave, and I. Karaman, Additively manufacturing nitinol as a solid-state phase change material, in 2020 19th IEEE Intersociety Conference on Thermal and Thermomechanical Phenomena in Electronic Systems (ITherm) (2020) pp. 821–826.

[53] D. J. Sharar, A. C. Leff, A. A. Wilson, and A. Smith, High-capacity high-power thermal energy storage using solid-solid martensitic transformations, Applied Thermal Engineering 187, 116490 (2021).

[54] Y. Tian, B. Hu, P. Dang, S. Dong, S. Sun, D. Xue, T. Lookman, and T. Zhang, Design of shape memory alloys with enhanced thermal management properties via adaptively constrained multi-objective optimization, Acta Materialia 306, 121874 (2026). https://doi.org/10.1016/j.actamat.2025.121874.
